# Integrated climate effects on nitrogen cycles in global grasslands

**DOI:** 10.1126/sciadv.aec5940

**Published:** 2026-05-06

**Authors:** Miao Zheng, Jinglan Cui, Luxi Cheng, Sitong Wang, Xiaoxi Wang, Xin Xu, Xiuming Zhang, Chen Wang, Chenchen Ren, Baojing Gu

**Affiliations:** ^1^ State Key Laboratory of Soil Pollution Control and Safety, Zhejiang University, Hangzhou 310058, China.; ^2^ College of Environmental and Resource Sciences, Zhejiang University, Hangzhou 310058, China.; ^3^ China Academy for Rural Development, Zhejiang University, Hangzhou, China.; ^4^ Department of Agricultural Economics and Management, School of Public Affairs, Zhejiang University, Hangzhou, China.; ^5^ Potsdam Institute for Climate Impact Research (PIK), Potsdam, Germany.; ^6^ International Institute for Applied Systems Analysis, Schlossplatz 1, A-2361 Laxenburg, Austria.; ^7^ Biosphere Sciences and Engineering, Carnegie Institution for Science, Stanford, CA, USA.; ^8^ Policy Simulation Laboratory, Zhejiang University, Hangzhou 310058, China.

## Abstract

Grasslands play a crucial role in providing essential ecosystem services through biogeochemical processes. Improving grassland productivity and nitrogen use efficiency, reducing reactive nitrogen losses, and ensuring environmental sustainability represent major challenges, especially under the influence of global climate change. While previous studies have shown substantial effects of individual climate change factors on grassland nitrogen cycling, a comprehensive understanding of how grassland nitrogen cycling responds to multiple climate change remains limited. In this study, using data from 150 countries, we identified climate warming as the primary driver of increased nitrogen harvest, biological nitrogen fixation, and nitrogen surplus in global managed and undisturbed grasslands. These increases, with respective increments of 19.8, 8.8, and 28.2%, were determined by comparing scenarios with and without climate change from 1980 to 2020. Precipitation variability further amplifies these nitrogen increases, displaying notable spatial heterogeneity. Conversely, elevated atmospheric CO_2_ levels mitigate nitrogen surplus by enhancing plant nitrogen uptake. Under the SSP2-RCP4.5 scenario for the year 2050, nitrogen input, harvest, and surplus in global grasslands are projected to increase annually by 22.3, 7.2, and 15.1 million tonnes, respectively, compared to baseline scenarios. These climate-induced alterations in nitrogen budgets could incur additional costs up to USD $69 billion because of associated impacts on human health and ecosystem integrity. Our findings emphasize the urgent need for robust management strategies aimed at mitigating the negative effects of climate change on grassland nitrogen cycling, thereby supporting global sustainable development objectives.

## Teaser

Grasslands respond to integrated climate effects, influencing nitrogen cycles under future climate change.

## INTRODUCTION

Climate change is accelerating at an unprecedented speed (*1*), including elevated atmospheric carbon dioxide (CO_2_) concentrations, global warming, and altered precipitation regimes. CO_2_ levels are projected to surpass 600 parts per million (ppm) by the end of the 21st century (*2*), with surface air temperature anticipated to rise by 1.0° to 3.7°C (*1*). Concurrently, global annual precipitation is forecasted to increase by ~4.6% (*1*, *3*), although this variation will exhibit considerable spatial variability. Northern high latitudes are expected to experience increased precipitation, while reductions are predicted for Europe, East Asia, South Asia, and parts of South America and Africa (*1*, *4*). These climatic shifts underscore the critical need for comprehensive studies to support the formulation of effective climate adaptation strategies.

Grasslands cover more than 40% of Earth’s land surface (*5*), including both managed and undisturbed grasslands, and their susceptibility to climate change is increasingly concerning (*6*). Experimental findings suggest that combined increases in temperature and CO_2_ concentrations can enhance grassland productivity (*7*, *8*), while in mesic and high-altitude grasslands, precipitation changes often exert greater influence on soil nitrogen (N) pools than warming alone (*9*). In addition, climate change is expected to exacerbate soil erosion and degradation, further affecting wilder rangelands (*10*). Although individual climatic impacts on grasslands have been examined (*11*–*17*), integrated climate effects require further investigation. In addition, substantial losses of reactive N (N_r_) pose environmental and health risks, including increased greenhouse gas emissions, biodiversity loss, and eutrophication (*18*, *19*). Prior research has typically omitted comprehensive evaluations of N dynamics across essential system compartments, creating notable uncertainties in global grassland N budget projections under climate change.

In this study, we assess how climate change, specifically variations in atmospheric CO_2_ levels, air temperature, total precipitation, and their interactions, influences N harvest, N surplus, biological N fixation (BNF), fertilizer, and manure in global both managed and undisturbed grasslands. Leveraging long-term panel data from more than 150 countries spanning from 1980 to 2020, we used a fixed-effects panel (FEP) regression model to control for temporal and cross-country biases. Structural equation modeling (SEM) enabled the elucidation of direct and indirect pathways by which climate change affects grassland N cycles. Furthermore, we used counterfactual analyses of historical data from 1980 to 2020 to determine the specific impacts of CO_2_, temperature, and precipitation changes on grassland N budgets. Projected future N budgets under various climate change scenarios were generated using the Model of Agricultural Production and its Impact on the Environment (MAgPIE) (*20*) and the Coupled Human and Nature System (CHANS) (*21*–*24*) models (fig. S1). These projections were benchmarked against baseline scenarios reflecting different Shared Socioeconomic Pathways (SSPs) and Representative Concentration Pathways (RCPs). Last, we conducted a cost-benefit analysis to quantify the economic implications of climate-driven alterations on ecosystems and human well-being (Materials and Methods).

## RESULTS AND DISCUSSION

### Impact of climate change

**Fig. 1.**
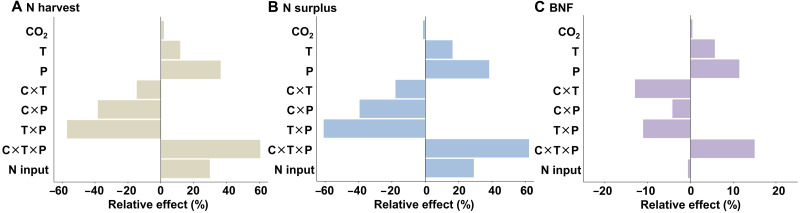
Relative effects of climate change on N harvest, N surplus, and biological N fixation. (**A**) Relative effects of CO_2_, temperature, precipitation, interactions, and N input on N harvest. (**B**) N surplus. (**C**) BNF. CO_2_, temperature, and precipitation are the mean CO_2_ concentrations (10^3^ ppm), mean annual temperature (10^2^°C), and mean annual precipitation (10^3^ mm), respectively; C×T, the interaction between CO_2_ and temperature (10^4^ ppm·°C); C×P, the interaction between CO_2_ and precipitation (10^5^ ppm·mm); T×P, the interaction between temperature and precipitation (10^3^°C·mm); C×T×P, the interaction among CO_2_, temperature, and precipitation (10^6^ ppm·°C·mm). The effects of each item were derived from the ratio of the standardization coefficient of each explanatory variable to the standard deviation of the explained variable covering all sample countries from 1980 to 2020. The coefficient refers to standardization coefficient based on models in Table 1.

Using an FEP regression model (Table 1), we analyzed standardized explanatory coefficients from 1980 to 2020 to evaluate the relative effects of climate change on N harvest, BNF, and N surplus in global grasslands (Fig. 1). CO_2_ concentrations were assigned using nearest-neighbor sampling on the basis of country boundaries and averaged monthly. Meanwhile, temperature and precipitation were spatially sampled within grassland distribution boundaries. Our findings indicate that temperature and precipitation significantly and positively influence these N indicators, with temperature effects following an inverted U-shaped pattern (Table 1). Variation in dominant grassland species, particularly the distinct responses between C_3_ and C_4_ grasslands, likely explains the differing temperature thresholds observed across countries (*17*). Conversely, elevated CO_2_ exhibited a relatively minor negative impact (−1.3%) on N surplus (table S1). Interaction effects between climatic factors predominantly yielded negative impacts. Specifically, the interaction effects between temperature and precipitation indicated that precipitation variability reduced the warming effect of all three N cycle indicators. This outcome can be attributed to the constrained adaptive capacity of grassland organisms under combined heat and drought conditions (*25*, *26*). Nevertheless, a positive compensatory effect emerged from the three-way interaction among CO_2_, temperature, and precipitation. Specifically, elevated CO_2_ enhanced water use efficiency through stomatal closure and increased root density, which may alleviate or even counterbalance heat and drought stress (*27*, *28*). As a result, this interaction led to relative increases of 60.3 and 62.0% for N harvest and N surplus, respectively (table S1).

**Table 1.** Impacts of climate change on N budgets. Note: Each column represents a separate regression model, i.e., the FEP model. Models 1, 2, 3, 4, and 5 present the regression results responding to N harvest, N surplus, BNF, fertilizer, and manure, respectively. Significance levels based on *P* values are indicated by asterisks. Standard errors (s.e.) are shown in parentheses. *N*, the number of samples. Each regression equation includes control variables: grassland area, ratio of organic and synthetic fertilizers, and ratio of biological fixation. All equations include country and year fixed effects.

	Ln N harvest (Tg)	Ln N surplus (Tg)	Ln BNF (Tg)	Ln fertilizer (Tg)	Ln manure (Tg)
	Model 1	Model 2	Model 3	Model 4	Model 5
CO_2_ (10^3^ ppm)	4.939*	−3.393	1.419	−23.411**	−6.110**
s.e.	(2.668)	(2.169)	(3.355)	(11.875)	(2.808)
Temperature (10^2^°C)	12.312***	14.427***	16.488***	−60.328***	−33.176***
s.e.	(2.182)	(1.774)	(2.730)	(9.578)	(1.896)
Temperature^2^ (10^2^°C)	−11.576**	−9.147**	−34.780***	−49.189*	18.930***
s.e.	(5.669)	(4.608)	(7.103)	(25.242)	(5.017)
Precipitation (10^3^ mm)	37.537***	37.326***	12.609	0.459	12.931**
s.e.	(5.112)	(4.157)	(6.429)	(22.803)	(5.080)
Precipitation^2^ (10^3^ mm)	−1.046	2.578	−4.048	−30.275**	4.640*
s.e.	(2.726)	(2.215)	(3.425)	(12.178)	(2.553)
CO_2_×temperature (10^4^ ppm·°C)	−2.690***	−3.173***	−2.987***	16.921***	7.257***
s.e.	(0.586)	(0.477)	(0.736)	(2.592)	(0.482)
CO_2_×precipitation (10^5^ ppm·mm)	−10.232***	−10.149***	−1.401	−1.074	−4.025***
s.e.	(1.334)	(1.085)	(1.677)	(5.951)	(1.331)
Temperature×precipitation (10^3^°C·mm)	−2.014***	−2.076***	−0.486*	2.904***	−0.248
s.e.	(0.224)	(0.182)	(0.281)	(0.994)	(0.211)
CO_2_×temperature×precipitation (10^6^ ppm·°C·mm)	5.548***	5.508***	1.713**	−6.012**	0.826
s.e.	(0.576)	(0.468)	(0.724)	(2.559)	(0.552)
Ln BNF (Tg)	0.243***	0.197***			
s.e.	(0.013)	(0.011)			
Ln fertilizer (Tg)	0.101***	0.101***	0.002		
s.e.	(0.004)	(0.003)	(0.005)		
Ln manure (Tg)	0.338***	0.348***	−0.025		
s.e.	(0.017)	(0.014)	(0.022)		
					
Country	Yes	Yes	Yes	Yes	Yes
Year	Yes	Yes	Yes	Yes	Yes
*N*	3811	3817	3817	3928	6581
Adj-*R^2^*	0.992	0.994	0.989	0.940	0.986

* *P* < 0.1.

** *P* < 0.05.

*** *P* < 0.01.

To quantify climate change impacts further, comparisons were made between scenarios with and without climate change (Materials and Methods). Temperature emerged as the dominant driver, responsible for annual increases of 19.8% in N harvest, 8.8% in BNF, and 28.2% in N surplus from 1980 to 2020 (fig. S2). Including precipitation alongside temperature slightly moderated these overall increments. Combined consideration of CO_2_, temperature, and precipitation yielded annual increases of 24.3% for N harvest, 10.0% for BNF, and 24.9% for N surplus, suggesting that CO_2_ may partly offset the rise in N surplus by enhancing plant N uptake. By 2020, ~79% of global grasslands simultaneously experienced increased N harvest, BNF, and N surplus, while declines occurred primarily in Canada and Russia (figs. S3 and S4). Consequently, regions such as the Latin America, Africa, Europe, Oceania, North America (except Canada), and most of Asia encountered rising N surplus, posing substantial sustainability challenges. Notably, spatial variation in precipitation within large countries like China and the United States suggests that using national averages might underestimate local effects on grassland N dynamics.

### Driving pathways

**Fig. 2.**
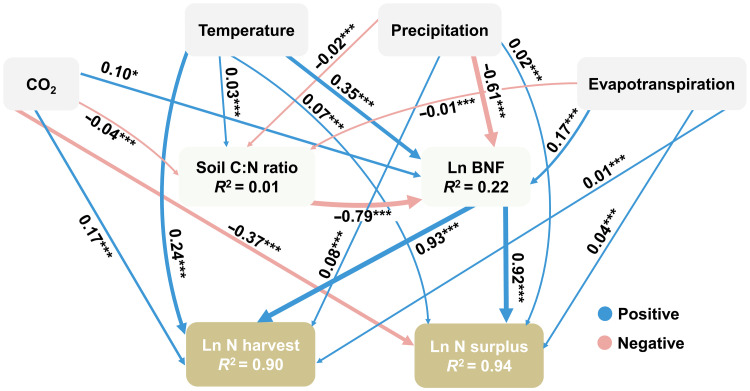
Impact pathways of climate changes on N harvest, N surplus, and biological N fixation. CO_2_, temperature, and precipitation are the mean CO_2_ (10^1^ ppm), mean annual temperature (10^1^°C), and mean annual precipitation (10^2^ mm) in 2020, respectively; evapotranspiration (10^2^ mm); soil C:N ratio, soil C content ratio divided by N content ratio (10^2^). Ln BNF is the logarithm of biological N fixation (Gg N), Ln N harvest is the logarithm of N harvest (Gg N), and Ln N surplus is the logarithm of N surplus (Gg N). Each number on the linear edges represents standardized path coefficients, indicating the extent to which the standard deviation of the dependent variable changes when each independent variable changes by one standard deviation. The blue and red lines represent positive and negative impacts, respectively. Significance levels based on *P* values are indicated by asterisks: *P < 0.1, **P < 0.01, and ***P < 0.001.

To comprehensively investigate how climate change affects N harvest, N surplus, and BNF, we applied SEM to examine both direct and indirect influence pathways (Fig. 2). The SEM integrated atmospheric CO_2_ concentration, temperature, precipitation, and evapotranspiration as driving variables, affecting N harvest and N surplus directly and indirectly through soil C:N ratios and BNF. While including interaction effects reduced model fit, their impacts have been analyzed in the panel analysis. Temperature exhibited the most substantial direct positive impact on N harvest, with a standardized coefficient of 0.24 (table S2), attributed to extended growing seasons under warming conditions (*29*, *30*). Elevated atmospheric CO_2_ levels were also significantly correlated with increased N harvest (0.17), reflecting its fertilization effect on photosynthesis and plant productivity (*31*, *32*). In contrast, precipitation and evapotranspiration had relatively minor direct effects, with coefficients of 0.08 and 0.01, respectively. BNF and soil C:N ratio were considered potential mediators, with soil C:N ratio exerting primarily indirect effects on N harvest and N surplus through its influence on BNF (*33*, *34*). Temperature exerted a strong positive indirect effect (0.33) on N harvest through enhanced BNF, primarily driven by increased microbial activity associated with N cycling (*35*). Indirect effects mediated by the soil C:N ratio were comparatively minor (Fig. 2). Consequently, standardized net effects of CO_2_, temperature, precipitation, and evapotranspiration on N harvest were 0.29, 0.54, −0.47, and 0.18, respectively (table S2).

Regarding N surplus, elevated CO_2_ exhibited a strong negative direct effect (−0.37), suggesting enhanced plant N uptake under elevated CO_2_ levels, thereby reducing residual soil N (*36*). Despite positive indirect effects through BNF and soil C:N ratio, the overall net effect remained negative (−0.25), indicating a mitigating influence of elevated CO_2_ on N losses (Fig. 2). Conversely, temperature displayed a positive net effect (0.37) on N surplus, predominantly through a strong indirect effect (0.32) associated with increased BNF driven by microbial activity under warming conditions (*29*, *30*). Precipitation and evapotranspiration contributed smaller positive direct effects, with coefficients of 0.02 and 0.04, respectively (table S3). These findings underscore the notable implications of climate-driven increases in N pollution for atmospheric quality, soil health, and aquatic ecosystems (*37*, *38*). Addressing these impacts necessitates comprehensive management strategies, including efficient feed management, fertilization practices, and manure management (*39*).

### Future scenarios

Integrating the CHANS model with MAgPIE, we assessed climate change impacts on grassland N budgets at a spatial resolution of 0.5° by 0.5°. This resolution optimizes the balance between capturing regional variability and computational efficiency for large-scale, long-term analyses. Projections were based on RCPs and SSPs (*1*), forming baseline and climate change scenarios reflecting diverse societal, economic, and environmental conditions (*40*). To assess future trends in grassland N budgets, we accounted for CO_2_, temperature, and precipitation changes in two scenarios (SSP1-RCP2.6, representing a “sustainable society,” and SSP2-RCP4.5, representing a “business-as-usual” scenario). These were compared to baseline scenarios, which did not include climate change effects such as warming, increased CO_2_, and altered precipitation patterns. Future climate data (2030 to 2050) at 0.5°-by-0.5° resolution were obtained from the CanESM5 model simulations under the Climate Model Intercomparison Project Phase 6 (CMIP6) (https://esgf-node.llnl.gov/projects/cmip6/) (fig. S5) (*1*).

Our projections reveal consistent changes in global grassland N cycles across climate scenarios (fig. S6). Total N inputs rise from the baseline SSP2 scenario (133.7 to 138.0 Tg N year^−1^) to the SSP2-4.5 scenario (151.4 to 160.3 Tg N year^−1^). A similar increase is observed from the SSP1 scenario (128.6 to 116.7 Tg N year^−1^) to the SSP1-2.6 scenario (151.1 to 161.1 Tg N year^−1^) over the two decades. Correspondingly, N harvest exhibited similar trends, rising from the baseline SSP2 scenario (92.3 to 95.1 Tg N year^−1^) to the SSP2-4.5 scenario (95.8 to 102.3 Tg N year^−1^). Likewise, projections show an increase from the SSP1 scenario (88.7 to 80.5 Tg N year^−1^) to the SSP1-2.6 scenario (101.0 to 106.7 Tg N year^−1^). N surplus also increases from the baseline SSP2 scenario (41.5 to 42.9 Tg N year^−1^) to the SSP2-4.5 scenario (55.6 to 58.0 Tg N year^−1^) and, similarly, from the SSP1 scenario (39.9 to 36.3 Tg N year^−1^) to the SSP1-2.6 scenario (50.1 to 54.4 Tg N year^−1^) over the same period (figs. S7 to S9).

**Fig. 3.**
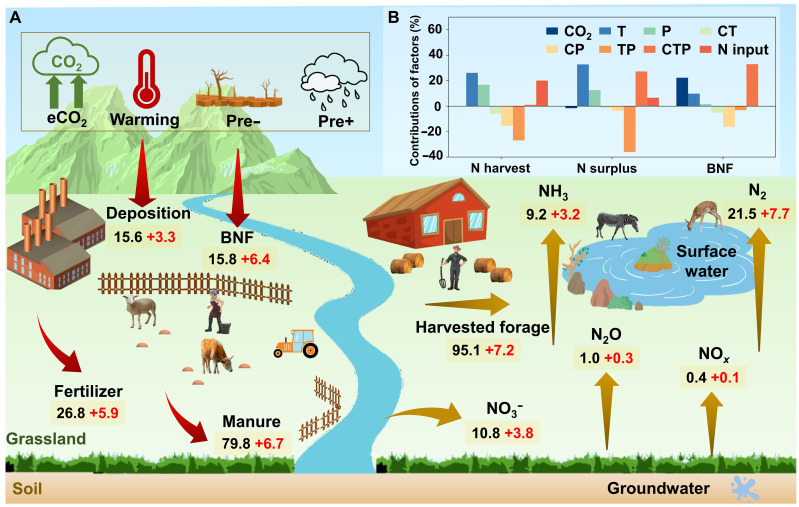
Projected changes in global grassland N budgets and contributions of climate drivers under the SSP2-4.5 scenario by 2050. (**A**) N flows in global grasslands under the climate change SSP2-4.5 scenario by 2050. The N fluxes, which include N input and N output, are shown by red and yellow arrows, respectively. The values of the N flows are shown in dark black for the grassland N budget in the baseline scenario in 2050 and in red for the climate change SSP2-4.5 scenario compared to the baseline scenario. These future simulated annual values (in Tg N year^−1^, projected to 2050) represent the integrated N cycle projections for both managed and undisturbed grasslands, as derived from the MAgPIE and CHANS models. ECO_2_, elevated CO_2_ concentration; Pre−, decreased precipitation; Pre+, increased precipitation. (**B**) Contributions of individual climatic factors to total climate impacts. T, temperature; P, precipitation; CT, the interaction between CO_2_ and temperature; CP, the interaction between CO_2_ and precipitation; TP, the interaction between temperature and precipitation; CTP, the interaction among CO_2_, temperature, and precipitation.

**Fig. 4.**
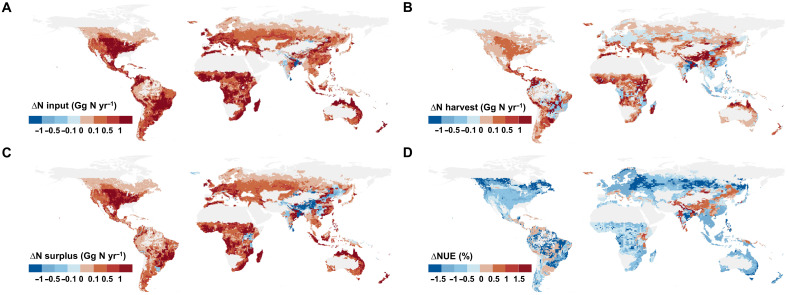
Spatiotemporal variations of global grassland N budget changes between the baseline scenario and the climate change SSP2-4.5 scenario in 2050. (**A**) ΔN input between the baseline scenario and the climate change SSP2-4.5 scenario in 2050; (**B**) ΔN harvest; (**C**) ΔN surplus; (**D**) ΔNUE. yr, year. Values in the legend demonstrate the average annual grassland N budget within a grid cell (0.5° by 0.5°). The base map is from GADM data, which are freely available for academic and other noncommercial use (https://gadm.org/).

Under the climate change SSP2-4.5 scenario for 2050, global grasslands are projected to experience notable increases in N input (22.3 Tg N year^−1^), N harvest (7.2 Tg N year^−1^), and N surplus (15.1 Tg N year^−1^) compared to the no-climate-change baseline (Fig. 3A and table S4). Relative to observed 2020 values, these changes correspond to increases of 16.8, 7.9, and 36.7%, respectively, with considerable regional variations (fig. S10). Regions experiencing notable increases in N harvest include northern Australia, Mexico, Colombia, Argentina, Europe (e.g., Italy and Spain), western China, and parts of Africa (e.g., Ethiopia and Sudan). Moderate gains are anticipated in the US, Russia, and eastern Australia, while declines are projected for Brazil, Tanzania, Congo, and Southeast Asian countries (Fig. 4B). Warming and precipitation changes notably influence N harvest, although their interactions partially offset potential gains (Fig. 3B). Globally, grassland N use efficiency (NUE) is expected to decline from 68.9 to 63.8% by 2050, with improvements in Latin America, Africa, and Asia regions but declines in most regions worldwide (Fig. 4D and fig. S10).

The projected increase in global N input by 2050 under the SSP2-4.5 scenario includes contributions from BNF (6.4 Tg N year^−1^), N deposition (3.3 Tg N year^−1^), fertilizer (5.9 Tg N year^−1^), and manure (6.7 Tg N year^−1^) (Fig. 3 and table S4), with substantial regional variability (fig. S11). Elevated CO_2_ primarily drives the BNF increase, contributing by 22.4%, thus boosting N input (Fig. 3B). Most global grasslands are expected to see increases in both N inputs and losses, particularly in North America, northern Australia, Brazil, and most Africa (Fig. 4A). Enhanced N surplus leads to greater atmospheric emissions of NH_3_ (3.2 Tg N year^−1^), N_2_O (0.3 Tg N year^−1^), NO*_x_* (0.1 Tg N year^−1^), and nonreactive N_2_ emissions to the atmosphere (7.7 Tg N year^−1^), alongside NO_3_^−^ losses to water systems (3.8 Tg N year^−1^) (Fig. 3 and fig. S12), raising concerns about eutrophication, especially in aquatic ecosystems (*41*). Key regions identified as N loss hotspots include North America, Latin America, Africa, northern Australia, and parts of Europe (Fig. 4C), emphasizing the need for targeted management practices to mitigate environmental impacts.

### Cost-benefit analysis

**Fig. 5.**
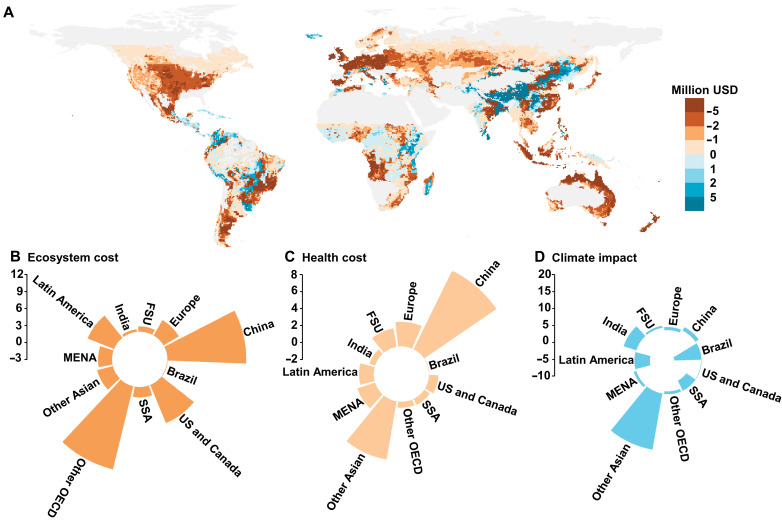
Cost-benefit analysis of climate change in global grasslands under the climate change SSP2-4.5 scenario compared to the baseline SSP2-4.5 scenario by 2050. (**A**) Maps display the cost-benefit analysis of climate changes in global grasslands. The legend values represent the cost-effectiveness from the average annual grassland N budget within each grid cell (0.5° by 0.5°). The base map is from GADM data, which are freely available for academic and other noncommercial use (https://gadm.org/). (**B**) Global ecosystem cost contributed by some grassland areas; (**C**) health cost; (**D**) climate impact. The positive brown values and negative blue values represent costs, while negative brown values and positive blue values represent benefits. The unit is billion USD. FSU, Former Soviet Union; MENA, Middle East and North Africa; SSA, Sub-Saharan Africa. Future climate change (CO_2_, temperature, and precipitation) levels under this scenario are obtained from CMIP6 model simulations.

Under the climate change SSP2-4.5 scenario for 2050, the projected cost-benefit analysis for global grasslands indicates an estimated loss of $75 billion US dollars and a gain of $6 billion US dollars, resulting in a net cost of $69 billion US dollars. This analysis includes costs and benefits associated with changes in ecosystem services, human health, and climate impacts (see the “Cost-benefit analysis” section). Regionally, most areas are expected to incur additional costs. The highest costs are projected for China (−$21 billion US dollars), other Organisation for Economic Cooperation and Development (OECD) countries (−$15 billion US dollars), Latin America (−$11 billion US dollars), Brazil (−$8 billion US dollars), US and Canada (−$7 billion US dollars), and Europe (−$6 billion US dollars) (Fig. 5A and fig. S13). The majority of these costs are attributable to ecosystem costs (−$48 billion US dollars) and human health costs (−$27 billion US dollars), primarily driven by increased N_r_ emissions resulting from climate change (Fig. 5, B and C). Conversely, the benefits primarily stem from climate impacts (+$6 billion US dollars), largely due to increased N harvest in grasslands (Fig. 5D). Notably, other Asian countries account for the largest share of global climate impact benefits (+$18 billion US dollars), while China bears the largest share of human health costs (−$9 billion US dollars). Other OECD countries contribute notably to global ecosystem costs (−$14 billion US dollars). While some regions may benefit from climate impacts, the widespread implementation of adaptation strategies is essential to protect ecosystems, human health, and ensure long-term global sustainability (*38*, *42*).

### Future perspective

Projected changes in integrated climate factors are expected to reshape the N cycle in global grasslands. Our results suggest that future climate changes may boost forage production, potentially enhancing livestock yields in Australia, US, Mexico, Colombia, Argentina, southern Europe, western China, and parts of Africa. However, these areas are also likely to experience increased N_r_ losses, which could degrade air quality, soil health, and water ecosystems (*43*, *44*). Meanwhile, regions such as Brazil, Tanzania, Congo, and Southeast Asian countries are also expected to face reduced productivity, posing challenges to meeting the rising food and protein demands of growing populations (*45*). To counter these negative effects, adopting sustainable and integrated management strategies is essential in managed systems. Enhancing grassland infrastructure and training, alongside improved technologies, can strengthen resilience to climate impacts (*46*). Increasing NUE and promoting sustainable grassland management are critical to moderating temperature increases and reducing N_r_ emissions from grasslands (*47*, *48*). Policy-makers should focus on optimizing fertilizer and manure management while strategically managing other N inputs such as deposition (*49*, *50*). In addition, improving forage quality and refining livestock feed formulas can lower the energy demands of feed production (*51*), thereby reducing N losses. Early and targeted action is critical to mitigating the future impacts of climate change on grasslands and achieving global sustainability goals (*52*, *53*).

Furthermore, enhancing the representation of the N cycle in land surface models within ESMs (Earth System Models) is essential for accurately assessing climate change impacts on carbon (C)-N interactions in grasslands (*54*, *55*). Although this study covers more than 150 countries and nearly four decades of data, its resolution is constrained to annual scales, limiting the ability to capture climatic heterogeneity, particularly in China, Russia, and the US. Variations in CO_2_, temperature, and precipitation across regions, as well as intra-annual fluctuations, are not explicitly accounted for. For instance, it remains unclear whether annual precipitation totals result from extreme events or are evenly distributed throughout the year. Moreover, arid and semiarid conditions can have an impact on NUE and N content within countries related to water colimitation, management, and varieties (*56*), which are not fully investigated. While this study includes both managed and undisturbed grasslands, it does not explicitly distinguish between them, and different management regimes may elicit distinct responses (*57*). Despite these limitations, this study advances a scientific understanding of the relationship between climate change and spatial variations in global grassland N budgets. Future research should explore additional methodologies, such as machine learning, to further validate climate change impacts (*58*). Beyond examined factors like CO_2_ levels, temperature, and precipitation, investigating the role of extreme weather events is increasingly urgent (*59*, *60*). Moreover, understanding how grasslands dynamically respond to multiple factors, including management practices and land-use changes, is a critical avenue for future research.

## MATERIALS AND METHODS

### Data source

On the basis of N mass balance, the CHANS model provides a comprehensive assessment of N cycling and fluxes, integrating human-nature interactions through 14 subsystems that track N flows, making it well suited for evaluating N cycle dynamics under changing environmental conditions (fig. S1) (*21*–*24*). The historical data for global grassland N budgets from 1980 to 2020 at the country level, including N harvest, N surplus, BNF, fertilizer, and manure, are derived from this model. The grassland subsystem in CHANS includes both managed and undisturbed grasslands, as reflected in the underlying activity data. Key data sources include FAOSTAT (Food and Agriculture Organization online statistical databases of the United Nations) and peer-reviewed literature indexed in Google Scholar, Scopus, and Web of Science. Grassland area is obtained from www.fao.org/faostat/en/#data/RL, which includes land permanently (≥5 years) used for herbaceous forage crops, whether cultivated or naturally occurring, thereby encompassing both managed and undisturbed grasslands. Grassland N inputs, including BNF, N deposition, fertilizer, and manure, are derived from published studies that also include both managed and natural grasslands (*61*–*66*). The global grassland N harvest dataset is calculated for the entire “Permanent meadows and pastures” area, following Bouwman *et al.* (*67*), and applied uniformly without differentiating between grassland types. N surplus, including NH_3_, N_2_O, and NO*_x_* fluxes, as well as N loss to water (NO_3_^−^ fluxes), is estimated using the CHANS model. The model incorporates emission factors and process-based parameters synthesized from published modeling studies that cover both managed and undisturbed grasslands (*68*–*74*). The modeled N_r_ fluxes were validated using national monitoring data, demonstrating a strong correlation for air (*R*^2^ > 0.7) and a moderate correlation for water (*R*^2^ ~ 0.5) (*23*).

Climatic data, including average air temperature, total precipitation, and atmospheric CO_2_ level, were analyzed for this study. Monthly temperature (°C) and precipitation (mm/month) from 1980 to 2020 were obtained from the Climatic Research Unit Gridded Time Series (CRU TS version 4.07) (*75*). Monthly CO_2_ concentrations (ppm) for the same period were sourced from the Goddard Earth Sciences Data and Information Services Center (https://disc.gsfc.nasa.gov/), the National Oceanic and Atmospheric Administration (https://gml.noaa.gov/ccgg/trends/gl_data.html), and the National Earth System Science Data Center (www.geodata.cn). CO_2_ data were assigned using nearest-neighbor sampling on the basis of country boundaries and averaged monthly. Temperature and precipitation data were spatially sampled according to grassland distribution boundaries. Annual mean temperature, CO_2_ concentration, and total precipitation were then calculated from these monthly datasets. Other climate variables, such as evapotranspiration (ET_0_), were derived from WorldClim version 2.07 (*76*), while soil data were acquired from GLDAS (NASA Global Land Data Assimilation System) (https://ldas.gsfc.nasa.gov/gldas/soils). Simulated counterfactual monthly climate data for grasslands at the country level, representing scenarios with and without climate change (*77*), were acquired from the Cornell Institute for Social and Economic Research (https://doi.org/10.6077/pfsd-0v93). The dataset includes monthly averages from seven general circulation models based on the “hist-nat” (without climate change) and “historical” (with climate change) scenarios. Further details on the scenario analysis are provided in the “Scenario analysis” section.

### Grassland N budget

Unlike previous studies using a single model, our research combined MAgPIE (*20*) outputs with CHANS (*21*–*24*) simulations to project future global N budgets for grasslands, improving the robustness of the analysis. These projections were conducted at a 0.5°-by-0.5° spatial resolution. MAgPIE, a global partial equilibrium land-use framework, integrates regional economic and biophysical data (https://rse.pik-potsdam.de/doc/magpie/4.3/). Both the CHANS model and MAgPIE aim to study large-scale, long-term global environmental changes and identify potential adaptation strategies. A notable difference between the two models is their spatial resolution: MAgPIE operates at a finer resolution of 0.5° by 0.5°, while CHANS uses country-level data. Incorporating MAgPIE outputs into the CHANS model enhances spatial resolution, allowing for more localized assessments (fig. S14). This integration is made possible by the shared mass balance principles in both models, ensuring the compatibility of N flow data.

The N budget for grasslands can be determined by calculating the N input ($N_{\text{input}}$), N harvest ($N_{\text{harvest}}$), N surplus ($N_{\text{surplus}}$), and NUE as follows

$$\sum_{1}^{k}N_{\text{input},x}=\sum_{1}^{k}N_{\text{harvest},x}+\sum_{1}^{k}N_{\text{surplus},x}$$ (1)

$${\text{NUE}}_{x}=N_{\text{harvest},x}/N_{\text{input},x}$$ (2)

$$N_{\text{input},x}=N_{\text{man},x}+N_{\text{fer},x}+N_{\text{dep},x}+N_{\text{BNF},x}$$ (3)

$$N_{\text{surplus},x}=N_{\text{gas},x}+N_{\text{water},x}$$ (4)

where $N_{\text{input},x}$ includes sources such as manure ($N_{\text{man},x}$), fertilizer ($N_{\text{fer},x}$), deposition ($N_{\text{dep},x}$), and BNF ($N_{\text{BNF},x}$); $N_{\text{harvest},x}$ refers to the N contained in the harvested forage for each grid *x*; and $N_{\text{surplus},x}$ encompasses N loss through gaseous emissions (NH_3_, NO*_x_*, N_2_O, and N_2_) ($N_{\text{gas},x}$) and losses to water via leaching and runoff (NO_3_^−^) ($N_{\text{water},x}$).

The input factor ($\psi_{\text{input},x}$) and loss factor ($\psi_{\text{surplus},x}$) are defined as follows

$$\psi_{\text{input},x}=N_{\text{input}\cdot \text{component},x}/N_{\text{input},x}$$ (5)

$$\psi_{\text{surplus},x}=N_{\text{surplus}\cdot \text{component},x}/N_{\text{surplus},x}$$ (6)

where $N_{\text{input}\cdot \text{component},x}$ comprises four components: $N_{\text{man},x}$, $N_{\text{fer},x}$, $N_{\text{dep},x}$, and $N_{\text{BNF},x}$; $N_{\text{surplus}\cdot \text{component},x}$ includes two components: $N_{\text{water},x}$ and $N_{\text{gas},x}$.

The reactive N ($N_{r,x}$) fluxes encompass NH_3_ ($N_{NH_{3},x}$), N_2_O ($N_{N_{2}O,x}$), and NO*_x_* fluxes ($N_{NO_{x},x}$), as well as N loss to water (NO_3_^−^ fluxes) ($N_{\text{water},x}$)

$$N_{r,x}=N_{NH_{3},x}+N_{N_{2}O,x}+N_{NO_{x},x}+N_{\text{water},x}$$ (7)

### Statistical analysis

To assess the impact of climate change on N harvest, N surplus, BNF, fertilizer, and manure, we performed a panel analysis using a FEP model. Country-level data from 1980 to 2020 were used to estimate Eq. 8

$$\begin{array}{c} \text{ln}Y_{i,t}\\ =\eta +\alpha \times C{O_{2}}_{i,t}+\beta_{1}\times T_{i,t}+\beta_{2}\times T_{i,t}^{2}+\gamma_{1}\times P_{i.t}+\gamma_{2}\times P_{i.t}^{2}+\delta_{1}\times CT_{i.t}\\ +\delta_{2}\times CP_{i.t}+\delta_{3}\times TP_{i,t}+\delta_{4}\times \text{CT}P_{i,t}+\sum_{n}\vartheta_{n}q_{ni,t}+\sigma_{i}+\varepsilon_{t}+\mu_{i,t} \end{array}$$ (8)

where the subscripts *i* and *t* represent the country and year, respectively. The dependent variable $Y_{i,t}$ includes N harvest, N surplus, BNF, fertilizer, and manure. CO_2_, *T*, and *P* denote the average CO_2_ level (10^3^ ppm), air temperature (10^2^°C), and total precipitation (10^3^mm) for the year, respectively, with *T*^2^ and *P*^2^ representing the quadratic terms. CO_2_, temperature, and precipitation are used as key meteorological indicators to capture the climate change effects in this study. *CT* refers to the interaction between CO_2_ and temperature (10^4^ ppm·°C); *CP* refers to the interaction between CO_2_ and precipitation (10^5^ ppm·mm); *TP* captures the interaction between temperature and precipitation (10^3^°C·mm), *CTP* indicates the interaction among CO_2_, temperature, and precipitation (10^6^ ppm·°C·mm). The control variables $q_{n}$ include the grassland area, ratio of organic and synthetic fertilizers, and ratio of biological fixation. η is the constant term, while $\sigma_{i}$, $\varepsilon_{t}$, and $\mu_{i,t}$ are error items. α, β, γ, δ, and ϑ are the coefficients to be estimated. The results from the fixed-effects regression model are presented in Table 1.

In our fixed-effects model, we incorporated country-specific intercepts $\sigma_{i}$ to control for time-invariant factors, unobserved factors (*78*, *79*), such as national policies on agricultural trade and grassland soil conditions, that could cause estimation bias. By accounting for these fixed effects, we aim to more accurately isolate the effects of our independent variables. In addition, we incorporated the term $\varepsilon_{t}$ to capture common temporal trends, such as global economic changes, that affect all countries. To improve the robustness of our model, we included several control variables: grassland area, the ratio of organic to synthetic fertilizers, and the ratio of biological fixation. Grassland area refers to the total land dedicated to grasslands in each country, including both natural and managed grasslands (10^3^ ha). The ratio of organic to synthetic fertilizers is calculated by dividing the N from organic fertilizers by that from synthetic fertilizers. Similarly, the biological fixation ratio is the proportion of N fixed biologically compared to the N added through synthetic fertilizers. By integrating these control variables, we aim to isolate the true relationships between the independent and dependent variables, offering a clearer understanding of how each factor affects the grassland N budget.

To isolate the effects of changes in CO_2_, temperature, and precipitation on grassland N budgets, both country-specific time-invariant controls and year-fixed effects were included. However, a limitation of these fixed effects is that they may absorb a substantial portion of the weather variation. Table S5 shows the *R*^2^ and the standard deviation of residual weather variance not captured by the fixed effects. For example, year-fixed effects retain a significant amount of temperature variance, whereas including country-fixed effects greatly reduces the remaining variation, suggesting that geographic factors explain most of the temperature variation. To account for clustering of errors and spurious correlation of observations across time, cluster-robust standard errors are used, which also can reduce errors resulting from spatial correlation (*80*–*82*). The results are summarized in Table 1, with detailed statistics in table S6. Furthermore, we used standardized coefficient estimates, enabling comparisons across models with different dependent and independent variables. These coefficients represent the change in the explained variable (in standard deviations) resulting from a one-standard-deviation change in the corresponding climatic factor (table S1). Robustness checks on the base model were also conducted, with further details available in the Supplementary Materials. All statistical analyses were performed using Stata 18.0.

### Impact quantification

We assessed the impact of temperature change, the impact of temperature and precipitation changes, and the combined effects of temperature, precipitation, and CO_2_ changes by subtracting the projected counterfactual N value (without climate change) from the observed N value (with climate change). The temperature and precipitation data, both with and without climate change, were averaged across seven general circulation models (*77*). Given that CO_2_ data were not available from this source, we assumed that the CO_2_ level remained constant at 1980 under the without-climate-change scenario, while under the with-climate-change scenario, the CO_2_ level followed the original trend. For country *i*, the impact was determined using Eq. 8.

Using the coefficients from Eq. 8, we calculated the differences in the temperature change (*T*)–related variables between the climate change–affected ($T_{i,t}^{\text{With}}$) and unaffected weather ($T_{i,t}^{\text{Without}}$) for each country as follows

$$∆\text{ln}Y_{i,t}^{T}=f\left(T_{i,t}^{\text{With}}\right)-f\left(T_{i,t}^{\text{Without}}\right)$$ (9)

Accordingly, the differences in temperature- and precipitation-related variables (*TP*) at the country level were then calculated as follows

$$∆\text{ln}Y_{i,t}^{\text{TP}}=f\left(T_{i,t}^{\text{With}},P_{i,t}^{\text{With}}\right)-f\left(T_{i,t}^{\text{Without}},P_{i,t}^{\text{Without}}\right)$$ (10)

Then, the differences in temperature-, precipitation-, and CO_2_-related variables (*TPC*) at the country level were then calculated as follows

$$\begin{array}{c} ∆\text{ln}Y_{i,t}^{\text{TPC}}=f\left(T_{i,t}^{\text{With}},P_{i,t}^{\text{With}},C{O_{2}}_{i,t}^{\text{With}}\right)\\ -f\left(T_{i,t}^{\text{Without}},P_{i,t}^{\text{Without}},C{O_{2}}_{i,t}^{\text{Without}}\right) \end{array}$$ (11)

where *f* is the function related to Eq. 8.

We then add the difference $∆\text{ln}Y_{i,t}$ to the observed value to obtain the counterfactual values

$$Y_{i,t}^{\text{Without}\cdot T}=\text{exp}\left(\text{ln}Y_{i,t}-∆\text{ln}Y_{i,t}^{T}\right)$$ (12)

$$Y_{i,t}^{\text{Without}\cdot \text{TP}}=\text{exp}\left(\text{ln}Y_{i,t}-∆\text{ln}Y_{i,t}^{\text{TP}}\right)$$ (13)

$$Y_{i,t}^{\text{Without}\cdot \text{TPC}}=\text{exp}\left(\text{ln}Y_{i,t}-∆\text{ln}Y_{i,t}^{\text{TPC}}\right)$$ (14)

Last, the impact for country *i* in year *t* is calculated as the difference between the observed and counterfactual values

$$\text{Impac}t_{i,t}^{T}=\frac{\left(Y_{i,t}-Y_{i,t}^{\text{Without}\cdot T}\right)}{Y_{i,t}}$$ (15)

$$\text{Impac}t_{i,t}^{\text{TP}}=\frac{\left(Y_{i,t}-Y_{i,t}^{\text{Without}\cdot \text{TP}}\right)}{Y_{i,t}}$$ (16)

$$\text{Impac}t_{i,t}^{\text{TPC}}=\frac{\left(Y_{i,t}-Y_{i,t}^{\text{Without}\cdot \text{TPC}}\right)}{Y_{i,t}}$$ (17)

Note that we first estimated the impacts on manure and fertilizer. Next, BNF was derived considering manure and fertilizer impacts. Then, N harvest and surplus were derived incorporating manure, fertilizer, and BNF impacts. The global weighted mean of country-level impacts from 1980 to 2020 is presented in fig. S2.

### Structural equation modeling

In this study, we applied SEM to investigate the direct and indirect impacts of climate change on grassland N harvest and N surplus. Unlike FEP, which can only assess relationships with a single dependent variable and lacks the ability to compare multiple models directly, SEM offers a more integrated approach. SEM allows for the simultaneous evaluation of both direct and indirect pathways through which climate change affects various N cycle variables. This capability to model complex relationships makes SEM particularly effective in understanding the broader impacts of climate change on the N cycle. By combining SEM with FEP, we were able to gain a more nuanced perspective on climate change effects and conduct a more comprehensive analysis of the data.

Figure 2 illustrates an SEM model depicting the pathways through which climate change influences N harvest in grasslands. The model was developed by integrating theoretical and empirical evidence, identifying indicators such as CO_2_, temperature, precipitation, evapotranspiration, soil C:N ratio, and BNF. We inferred the relationships between these variables and constructed an initial model that included all indicators and subvariables. The model was then optimized on the basis of statistical significance and multiple fit indices, including the chi-squared test (*P* > 0.05), comparative fit index (CFI) >0.90, root mean square error of approximation (RMSEA) <0.05, standardized root mean square residual (SRMR) <0.08, and overall *R*^2^. Nonsignificant paths were removed to improve model interpretability. The final model, based on 2020 data, showed satisfactory fit indices: chi-squared test (*P* > 0.05), CFI > 0.90, RMSEA < 0.05, and SRMR < 0.08. The model’s overall *R*^2^ value is 0.33. Figure 2 also presents a similar SEM model for N surplus in grasslands, with the construction process following the same methodology as for the N harvest SEM model. The goodness-of-fit statistics for this model also fall within the expected range: chi-squared test (*P* > 0.05), CFI > 0.90, RMSEA < 0.05, and SRMR < 0.08, with an overall *R*^2^ value of 0.40 (Fig. 2).

### Scenario analysis

To assess the impacts of projected climate changes on the grassland N budget, we developed two scenarios, each with two subscenarios representing different SSPs and RCPs (fig. S5A). SSP scenarios explore potential changes in society, population, and the economy over the next century. Socioeconomic data related to these scenarios can be accessed through the SSP database at https://tntcat.iiasa.ac.at/SspDb. The RCPs represent likely future trajectories of greenhouse gas and aerosol emissions, driven by land-use changes (https://luh.umd.edu/data.shtml). SSP-RCP scenarios combine these socioeconomic and environmental pathways to project future outcomes. The baseline scenario assumes that CO_2_, temperature, and precipitation remain constant at 2020 levels. On the basis of the framework of Popp *et al.* (*40*) for predicting grassland area changes, which integrates dietary patterns, economic factors, and biophysical models, grassland N budgets under the baseline scenarios are projected every decade from 2030 to 2050 (*40*). In the climate change scenario, we incorporate projected shifts in CO_2_ concentrations, temperature, and precipitation using RCPs like RCP2.6 and RCP4.5 (fig. S5, B to D). Future climate data (2030 to 2050) are sourced from the CanESM5 model simulations under the World Climate Research Programme’s CMIP6 (https://esgf-node.llnl.gov/projects/cmip6/).

Then, the N harvest, N surplus, BNF, fertilizer, and manure trends under the climate change scenarios were calculated as follows

$$\begin{array}{c} ∆\text{ln}Y_{v,t}^{\text{climate}}=f\left(T_{i,t}^{\text{climate}},P_{i,t}^{\text{climate}},C{O_{2}}_{i,t}^{\text{climate}}\right)\\ -f\left(T_{i,t}^{\text{baseline}},P_{i,t}^{\text{baseline}},C{O_{2}}_{i,t}^{\text{baseline}}\right) \end{array}$$ (18)

$$Y_{v,t}^{\text{baseline}}=Y_{v,2020}^{\text{Observed}}\times \frac{\text{Are}a_{t,y}}{\text{Are}a_{2020,y}}$$ (19)

$$Y_{v,t}^{\text{climate}}=\text{exp}\left(\text{ln}Y_{v,t}^{\text{baseline}}+∆\text{ln}Y_{v,t}^{\text{climate}}\right)$$ (20)

$$\text{Chang}e_{v,t}^{\text{climate}}=Y_{v,t}^{\text{climate}}-Y_{v,t}^{\text{baseline}}$$ (21)

$$\text{Impac}t_{v,t}^{\text{climate}}=\frac{Y_{v,t}^{\text{climate}}-Y_{v,t}^{\text{baseline}}}{Y_{v,t}^{\text{baseline}}}$$ (22)

where *f* is the function related to Eq. 8. The log-transformed difference related to temperature, precipitation, and CO_2_ changes can be derived using the baseline temperature ($T_{i,t}^{\text{baseline}}$), precipitation ($P_{i,t}^{\text{baseline}}$), and CO_2_ ($C{O_{2}}_{i,t}^{\text{baseline}}$) and the temperature ($T_{i,t}^{\text{climate}}$), precipitation ($P_{i,t}^{\text{climate}}$), and CO_2_ ($C{O_{2}}_{i,t}^{\text{climate}}$) under climate change scenarios. *v* and *t* refer to the grid and year, respectively. Δ denotes the difference in values between the climate change and baseline scenarios in year *t*; $Y_{v,t}^{\text{baseline}}$ represents the grassland N budgets under the baseline scenarios for grid *v* in year *t*; $Y_{v,t}^{\text{climate}}$ refers to the simulated N values of N harvest, N surplus, BNF, fertilizer, and manure under the climate change scenarios for grid *v* in year *t*; $\text{Are}a_{T,y}$ and $\text{Are}a_{2020,y}$ denote the pasture area for the periods 2030 to 2050 and 2020, respectively, within each baseline scenario; and *y* signifies various regions, including Asia (Asian countries except the Middle East, Japan, and Former Soviet Union states), REF (reforming economies of Eastern Europe and the Former Soviet Union), LAM (countries of Latin America and the Caribbean), OECD (OECD 90 countries), and MAF (countries of the Middle East and Africa) (*40*).

Similarly, we first estimated the manure and fertilizer. Next, BNF was derived considering the manure and fertilizer impacts. Subsequently, N harvest and surplus were derived incorporating manure, fertilizer, and BNF impacts. Other N budgets were estimated using the following equation, adhering to the principle of N mass balance.

The values of N input and NUE under the climate change scenarios for grid *v* are calculated as follows

$$N_{\text{input},v,t}^{\text{climate}}=N_{\text{harvest},v,t}^{\text{climate}}+N_{\text{surplus},v,t}^{\text{climate}}$$ (23)

$$\text{NU}E_{v,t}^{\text{climate}}=\frac{N_{\text{harvest},v,t}^{\text{climate}}}{N_{\text{input},v,t}^{\text{climate}}}$$ (24)

where $N_{\text{input},v,t}^{\text{climate}}$ and $\text{NU}E_{v,t}^{\text{climate}}$ represent the N input and NUE under the climate change scenarios for grid *v* in year *t*, respectively.

In the climate change scenario, BNF ($N_{\text{BNF},v,t}^{\text{climate}}$), fertilizer ($N_{\text{fer},v,t}^{\text{climate}}$), and manure ($N_{\text{man},v,t}^{\text{climate}}$) were derived using Eqs. 18 to 20. The effects of climate changes on N deposition for grid *v* are calculated as follows

$$N_{\text{dep},v,t}^{\text{climate}}=N_{\text{input},v,t}^{\text{climate}}-N_{\text{BNF},v,t}^{\text{climate}}-N_{\text{fer},v,t}^{\text{climate}}-N_{\text{man},v,t}^{\text{climate}}$$ (25)

where $N_{\text{dep},v,t}^{\text{climate}}$ represents the N deposition under the climate change scenarios for grid *v* in year *t*.

Meanwhile, we assume that $\psi_{\text{surplus},w,v,t}$ remains unchanged, consistent with the 2020 observed levels.

$$\psi_{\text{surplus},w,v,2020}=\frac{N_{\text{surplus},w,v,2020}}{N_{\text{surplus},v,2020}}$$ (26)

$$N_{\text{surplus},w,v,t}^{\text{climate}}=N_{\text{surplus},v,t}^{\text{climate}}\times \psi_{\text{surplus},w,v,2020}$$ (27)

where $N_{\text{surplus},w,v,t}^{\text{climate}}$ represents the N surplus components under the climate change scenarios for grid *v* in year *t*, *w* includes N loss through gaseous emissions (NH_3_, NO*_x_*, N_2_O, and N_2_) and losses to water via leaching and runoff (NO_3_^−^), $N_{\text{surplus},v,t}^{\text{climate}}$ denotes the N surplus derived from Eqs. 18 to 20 under the climate change scenarios for grid *v* in year *t*, $N_{\text{surplus},v,2020}^{\text{climate}}$ is the observed N surplus for grid *v* in 2020, and $N_{\text{surplus},w,v,2020}^{\text{climate}}$ represents the observed N surplus components for grid *v* in 2020.

The overall effect of climate change is the combined effects of elevated CO_2_ concentrations, warming, and altered precipitation regimes. We attribute the integrated effect of climate change to individual factors. The individual contributions from factor *j* ($F_{j}$), including CO_2_ (*j*_1_), temperature (*j*_2_), precipitation (*j*_3_), the interaction between CO_2_ and temperature (*j*_4_), the interaction between CO_2_ and precipitation (*j*_5_), the interaction between temperature and precipitation (*j*_6_), and the interaction among CO_2_, temperature, and precipitation (*j*_7_) were calculated using the following equation

$$F_{j,t}=\frac{\sum_{v=1}^{N}∆Y_{v,j,t}^{\text{climate}}}{\sum_{v=1}^{N}Y_{v,t}^{\text{baseline}}}\times 100\%$$ (28)

N input (*j*_8_) was calculated as follows

$$F_{8,t}=\frac{\left(\sum_{v=1}^{N}∆Y_{v,t}^{\text{climate}}-\sum_{v=1,j=1}^{N,7}∆Y_{v,j,t}^{\text{climate}}\right)}{\sum_{v=1}^{N}Y_{v,t}^{\text{baseline}}}\times 100\%$$ (29)

where the subscripts *v* and *t* refer to the grid and year, respectively. Δ denotes the difference in values between the climate change and baseline scenarios in year *t*, $Y_{v,t}^{\text{baseline}}$ represents the grassland N budgets under the baseline scenarios for grid *v* in year *t*, and $Y_{v,t}^{\text{climate}}$ refers to the simulated N values of N harvest, N surplus, and BNF under the climate change scenarios for grid *v* in year *t*.

The physical changes in N budgets under the climate change SSP1-2.6 and SSP2-4.5 scenarios, relative to the baseline SSP1 and SSP2 scenarios, for 2050 are presented in Fig. 4 and figs. S7, S11, and S12. The relative changes for these scenarios compared to the baseline scenarios are shown in figs. S8 and S10. The contributions of individual factors are presented in Fig. 3B and fig. S9.

### Cost-benefit analysis

The cost-benefit analysis was conducted on the basis of model simulations and global N budget data to assess the effects of climate change scenarios on global grasslands, comparing them with baseline scenarios. Countries were categorized into groups, and monetary evaluations were performed at a 0.5°-by-0.5° grid scale, which were then extrapolated to regional and global levels (*24*). In the CHANS model, the projected costs and benefits were explicitly assigned to grasslands, reflecting their distinct N and C cycling characteristics. Because grasslands depend largely on BNF rather than synthetic fertilizers, they are more susceptible to shifts in natural N inputs under climate change. We also accounted for the heterogeneous distribution of N losses, including leaching and gaseous emissions, which impose disproportionate environmental and economic burdens on grasslands because of heightened pollution risks and biodiversity impacts. The analysis quantifies the costs and benefits of changes in N pollution and N harvest, calculating the monetized values of climate changes. The costs and benefits ($T_{\text{CBA}}$) are defined as the total increase in damage costs and benefits resulting from climate change, including ecosystem costs ($T_{\text{eco}}$), human health costs ($T_{\text{human}}$), and climate impacts ($T_{\text{climate}}$), with all values expressed in constant 2020 USD. These analyses are consistent with previous studies (*24*, *83*–*85*). The costs and benefits are calculated using the following equation

$$T_{\text{CBA}}=\sum_{1}^{x}\left(T_{\text{eco},x}+T_{\text{human},x}+T_{\text{climate},x}\right)$$ (30)

Damage costs are derived by multiplying the simulated change in N_r_ fluxes by a spatially and temporally adjusted unit damage cost ($U_{r,x}$). To improve the precision of damage cost estimates and account for future socioeconomic changes, the unit damage cost for ecosystem and human health *r* in grid *x* for the future year (2050) is adjusted from a reference year 2020 using projected changes in per capita gross domestic product (PGDP) and emission/population density (Density). This adjustment reflects changes in willingness to pay (WTP) and exposure risk, respectively.

The adjusted unit damage cost ($U_{r,x}$) is calculated as

$$U_{r,x,\text{climate}}=U_{r,x,\text{baseline}}\times \sqrt[{\alpha_{r}}]{\frac{{\text{PGDP}}_{2050,x}}{{\text{PGDP}}_{2020,x}}}\times \left(\frac{{\text{Density}}_{2050,x}}{{\text{Density}}_{2020,x}}\right)$$ (31)

where $U_{r,x,\text{climate}}$ and $U_{r,x,\text{baseline}}$ are the unit damage costs (USD per unit mass of pollutant) in grid *x* for 2050 under the climate change and baseline scenarios, respectively. The reference cost is obtained from the literature on the N-share method (human health costs) and WTP-based approaches (ecosystem costs). ${\text{PGDP}}_{2050,x}$ and ${\text{PGDP}}_{2020,x}$ are the projected year (2050) and reference year (2020)’s per capita gross domestic product, respectively. ${\text{Density}}_{2050,x}$ and ${\text{Density}}_{2020,x}$ are the projected year and reference year’s emission or population density, respectively. $\alpha_{r}$ is the adjustment coefficient specific to damage types, including ecosystem and human health costs, determined by fitting historical data on damage costs to socioeconomic factors.

Ecosystem costs represent the economic value of negative effects on ecosystem services resulting from changes in N_r_ losses (${\text{Flux}}_{x}$), which cause eutrophication, acidification, and biodiversity loss. The ecosystem cost ($T_{\text{eco}.x}$) for each grid *x* is calculated as follows

$$T_{\text{eco},x}={\text{Flux}}_{x,\text{climate}}\times U_{\text{eco},x,\text{climate}}-{\text{Flux}}_{x,\text{baseline}}\times U_{\text{eco},x,\text{baseline}}$$ (32)

where ${\text{Flux}}_{x,\text{climate}}$ and ${\text{Flux}}_{x,\text{baseline}}$ represent the N_r_ fluxes under the climate change and baseline scenarios for grid *x*, respectively, encompassing NO_3_^−^, N_2_O, NO*_x_*, and NH_3_ flows; and $U_{\text{eco},x,\text{climate}}$ and $U_{\text{eco},x,\text{baseline}}$ refer to the adjusted unit ecosystem damage costs associated with N_r_ loss under the climate change and baseline scenarios for grid *x*, respectively, estimated using the WTP-based approaches (*86*, *87*). To ensure the comparability of global ecosystem costs, the United States–based ecosystem damage costs resulting from N_r_ losses are adjusted for different regions on the basis of local WTP and purchasing power parity (*88*). While numerous cost-benefit studies have assessed N_r_ impacts on ecosystems in the United States and Europe (*85*), data for other regions remain limited.

Human health costs ($T_{\text{human},x}$) represent the economic impacts of health damages resulting from changes in N_r_ losses (${\text{Flux}}_{x}$) under projected climate scenarios (*86*). The costs are quantified using the following equation

$$T_{\text{human},x}={\text{Flux}}_{x,\text{climate}}\times U_{\text{human},x,\text{climate}}-{\text{Flux}}_{x,\text{baseline}}\times U_{\text{human},x,\text{baseline}}$$ (33)

where ${\text{Flux}}_{x,\text{climate}}$ and ${\text{Flux}}_{x,\text{baseline}}$ represent the N_r_ fluxes under the climate change and baseline scenarios for grid *x*, respectively, particularly NH_3_ and NO*_x_*; $U_{\text{human},x,\text{climate}}$ and $U_{\text{human},x,\text{baseline}}$ refer to the adjusted unit human health damage costs associated with N_r_ loss under the climate change and baseline scenarios for grid *x*, respectively, estimated using the N-share metric for PM_2.5_ (fine particulate matter) pollution, which models PM_2.5_ concentrations with and without N_r_ losses to evaluate their contribution (*89*).

Three key factors are essential for assessing the climatic impact ($T_{\text{climate},x}$) of climate changes on grasslands: C sequestration ($T_{\text{ecc},x}$), oxygen release ($T_{\text{eov},x}$), and N_r_ losses associated with climate change ($T_{N_{r},x}$) (*90*). Changes in N harvest were converted to the value of C sequestration and oxygen release using the replacement cost method. N_2_O, a potent greenhouse gas, has a substantial negative effect on the climate (*91*), while NO*_x_* and NH_3_ serve as aerosol precursors, reflecting long-wave solar radiation and contributing to a cooling effect on the climate system (*92*). The cost-benefit analysis of climate impact is therefore conducted as follows

$$T_{\text{climate},x}=T_{\text{ecc},x}+T_{\text{eov},x}+T_{N_{r},x}$$ (34)

$$T_{\text{ecc},x}=1.63\times ∆N_{\text{harvest},x}^{\text{climate}-\text{baseline}}\times \text{Are}a_{x}\times P_{c}$$ (35)

$$T_{\text{eov},x}=1.2\times ∆N_{\text{harvest},x}^{\text{climate}-\text{baseline}}\times \text{Are}a_{x}\times P_{O_{2}}$$ (36)

$$T_{N_{r},x}=∆N_{r,x}\times P_{N_{r},x}$$ (37)

where $T_{\text{ecc},x}$, $T_{\text{eov},x}$, and $T_{N_{r},x}$ represent the values of C sequestration, oxygen release, and N_r_ in grasslands for grid *x*, respectively; the constants 1.63 and 1.2 are fixed parameters (*93*); $∆N_{\text{harvest},x}^{\text{climate}-\text{baseline}}$ denotes the changes in N harvest under climate change scenarios compared to baseline scenarios for grid *x*; $∆N_{r,x}$ represents the N_r_ changes for grid *x*; $\text{Are}a_{x}$ indicates the forage harvest area; $P_{N_{r},x}$ is the monetary value of the climate impacts resulting from N_r_ losses in grid *x*, expressed in USD per kg N; and $P_{c}$ and $P_{O_{2}}$ are the prices of C sequestration and industrial oxygen (*94*–*96*), respectively, in USD per kg N. The industrial oxygen price is used as a proxy for the value of released oxygen, as its precise value is difficult to determine.

## Supplementary Materials

### The PDF file includes:

Supplementary Text Figs. S1 to S14 Tables S1 to S7 Legend for data S1 References
