# Supplementary material for: Integrated climate effects on nitrogen cycles in global grasslands
Source: Sci Adv. 2026 May 6;12(19):eaec5940. doi: 10.1126/sciadv.aec5940 (PMC13148316; doi:10.1126/sciadv.aec5940)
Supplement: Supplementary file 1 — Supplementary Text Figs. S1 to S14 Tables S1 to S7 Legend for data S1 References [file sciadv.aec5940_sm.pdf]

Science Advances
AAAS

Supplementary Materials for

Integrated climate effects on nitrogen cycles in global grasslands

Miao Zheng *et al.*

Corresponding author: Baojing Gu, bjgu@zju.edu.cn

Sci. Adv. **12**, eaec5940 (2026)
DOI: 10.1126/sciadv.aec5940

The PDF file includes:

Supplementary Text
Figs. S1 to S14
Tables S1 to S7
Legend for data S1
References

Other Supplementary Material for this manuscript includes the following:

Data S1

Supplementary Text

CHANS model

The Coupled Human and Natural System (CHANS) framework provides a comprehensive approach to examining interactions between human activities and natural processes(*97*). Originally designed to assess reactive nitrogen (N_r) production, environmental losses, and transfers across subsystems in China, CHANS has since been expanded globally through CHANS-Globe to analyze N budgets on an international scale(*21*). Grounded in N mass balance principles, the model maintains equilibrium across subsystems and the entire system. Recognized by the United Nations Environment Programme's International Nitrogen Management System (UNEP-INMS) as a crucial tool for studying the global N cycle, CHANS has been applied across diverse regions, including Japan, South Korea, Vietnam, Germany, Spain, United States, Canada, Brazil, Uganda, Kenya, and Australia.

1) Structure of the CHANS model

CHANS organizes N flows into four key functional components (Fig. S1):

1. **N-Processor** – Converts N inputs into agricultural and biological products, encompassing cropland, grassland, forest, livestock, industry, urban green spaces, and aquaculture.
2. **N-Remover** – Manages N waste through solid waste treatment and wastewater treatment systems.
3. **N-Consumer** – Represents N-consuming systems, including human and pet subsystems.
4. **Life-Supporter** – Maintains ecological balance by integrating surface water, groundwater, and the near-surface atmosphere(*97*).

By incorporating all N_r flows and interactions, CHANS calculates N inputs and outputs using a **mass-balance framework**, ensuring accurate tracking at both subsystem and system-wide levels(*21*, *22*). This study focuses on the grassland subsystem, utilizing data from national databases, FAO statistics, and peer-reviewed research. Key variables include population dynamics, economic activities, land use patterns, and fertilizer applications. CHANS facilitates N balance assessments by compiling data on N inputs, harvests, and surpluses. Additionally, human activities interact with N variables, generating region-specific N budget outputs. The model's temporal framework allows for historical, current, and projected N flow analyses.

2) Capabilities and limitations of CHANS model

CHANS demonstrates notable strengths, including its interdisciplinary integration, scenario-based simulations, diverse data sources, spatial specificity, and transparency. The model connects social, ecological, and biogeochemical processes, enabling a holistic assessment of how human activities influence N dynamics. By allowing multiple scenario simulations under varying socio-economic and environmental conditions, CHANS supports informed decision-making and policy development. Its reliance on a diverse range of data sources—including national statistics, global databases, and validated field observations—enhances the accuracy of its N flow estimations. The model is applicable at various spatial scales, from watersheds to global regions, making it suitable for both localized case studies and large-scale assessments. Furthermore, CHANS prioritizes transparency and accessibility, with all assumptions and N mass-balance calculations publicly available. Despite these strengths, CHANS has inherent limitations. Its simplified representation of complex social-ecological systems may not fully capture the intricacies of ecological processes and human behaviors.

To support continued research, a streamlined version of the CHANS model is publicly available for download at https://person.zju.edu.cn/en/bjgu, ensuring that researchers and policymakers can access a robust framework for studying N dynamics.

MAgPIE model

The Model of Agricultural Production and its Impact on the Environment (MAgPIE) is a global land-use framework that combines regional economic dynamics with biophysical processes(*20*). With a spatial resolution of 0.5 × 0.5 degrees, MAgPIE delivers precise projections of land-use patterns, yields, and total agricultural production costs per grid cell. Aligned with the Shared Socioeconomic Pathways (SSPs)(*98*), the model supports scenario-based simulations, significantly contributing to global evaluations, such as those featured in Intergovernmental Panel on Climate Change (IPCC) assessments(*1*). MAgPIE is widely applied to quantify terrestrial greenhouse gas emissions and explore key land-use sector dynamics, including interregional trade, agricultural productivity, and land-use transitions. These analyses address sustainable development challenges, particularly under future scenarios of increasing food demand and climate change impacts(*99*). For comprehensive details on MAgPIE's documentation, current initiatives, and accessible datasets, refer to the MAgPIE website at https://rse.pik-potsdam.de/doc/magpie/4.3/.

Robustness checks

Given that carbon dioxide (CO_2) concentration serves as a key climate change factor, we incorporated the quadratic term of annual CO_2 levels (ppm) into our analysis for a comprehensive validation. Using country-level data spanning 1980 to 2020, we formulated and evaluated the following equation:

$$lnY_{i,t} = \eta + \alpha_1 \times CO2_{i,t} + \alpha_2 \times CO2^2_{i,t} + \beta_1 \times T_{i,t} + \beta_2 \times T^2_{i,t} + \gamma_1 \times P_{i.t} + \gamma_2 \times P^2_{i.t} + \delta_1 \times CT_{i.t} + \delta_2 \times CP_{i.t} + \delta_3 \times TP_{i,t} + \delta_4 \times CTP_{i,t} + \sum_n \vartheta_n q_{ni,t} + \sigma_i + \varepsilon_t + \mu_{i,t} \quad [1]$$

Where the subscripts i and t represent the country and year, respectively. The dependent variable $Y_{i,t}$ includes N harvest, N surplus, BNF, fertilizer, and manure. CO_2, T and P denote the average CO_2 level (10^3 ppm), air temperature (10^2 °C) and total precipitation (10^3 mm) for the year, with $CO_2{}^2$, T^2 and P^2 representing the quadratic terms. *CT*, the interaction between CO_2 and temperature (10^4 ppm*°C); *CP*, the interaction between CO_2 and precipitation (10^5 ppm*mm); *TP* captures the interaction between temperature and precipitation (10^3 °C*mm), *CTP*, the interaction among CO_2, temperature, and precipitation (10^6 ppm*°C*mm). The control variables q_n include the grassland area, ratio of organic and synthetic fertilizers, and ratio of biological N fixation. η is the constant term, while σ_i, ε_t, and $\mu_{i,t}$ are error items. α, β, γ, δ, and ϑ are the coefficients to be estimated. The fixed-effects regression outcomes are detailed in Table S7, with descriptive statistics provided in Table S6. Findings from Table S5 indicate that temporal variations predominantly drive CO_2 fluctuations. Despite some changes in statistical significance, the results maintain overall robustness.

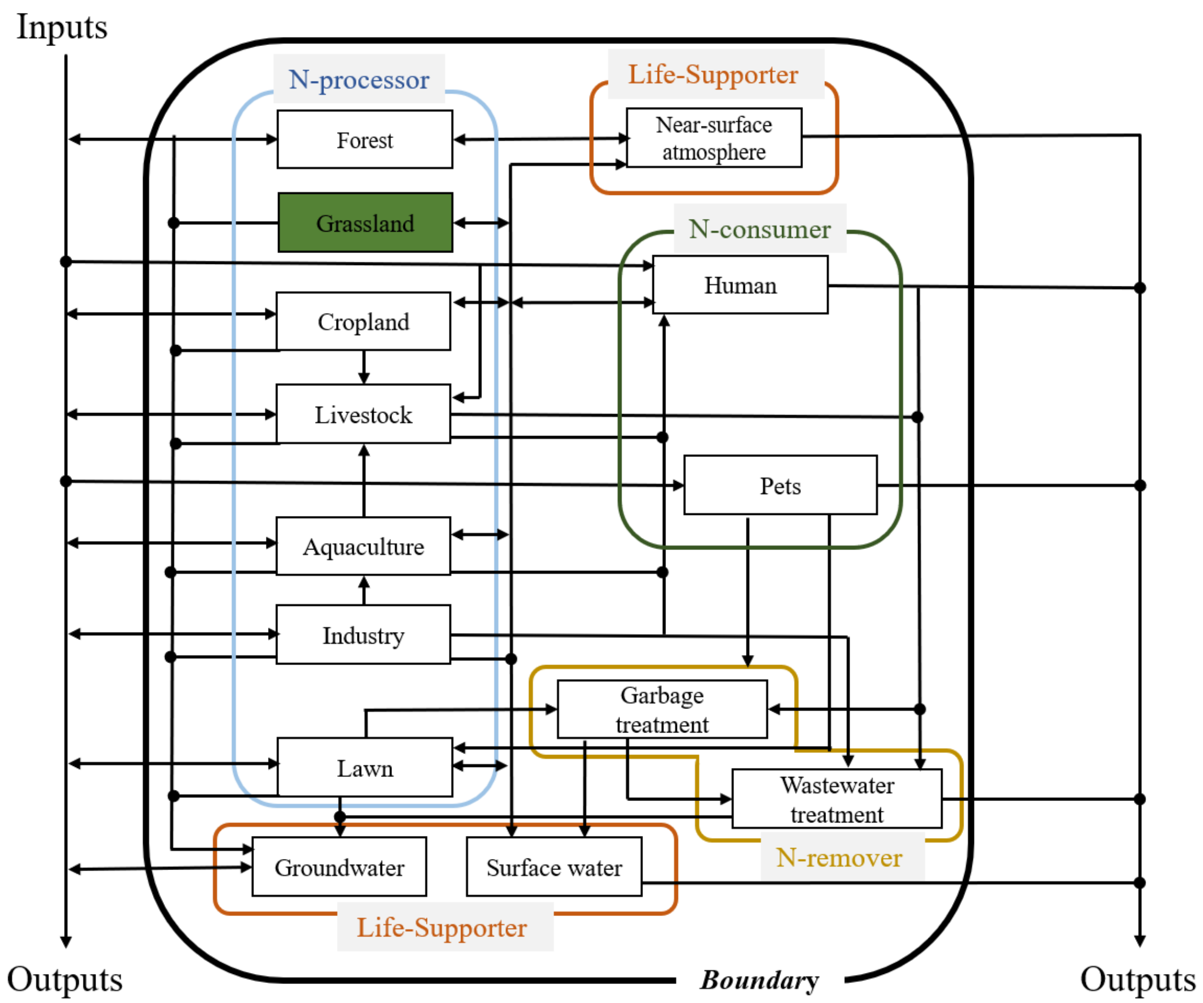

Fig. S1 Overview of the CHANS model. The CHANS model contains 14 subsystems that are divided into four functional groups: N-processors (indicated by a blue outline), N-consumers (green outline), N-removers (yellow outline), and life-supporters (orange outline). The focus of this study is on the grassland sub-system.

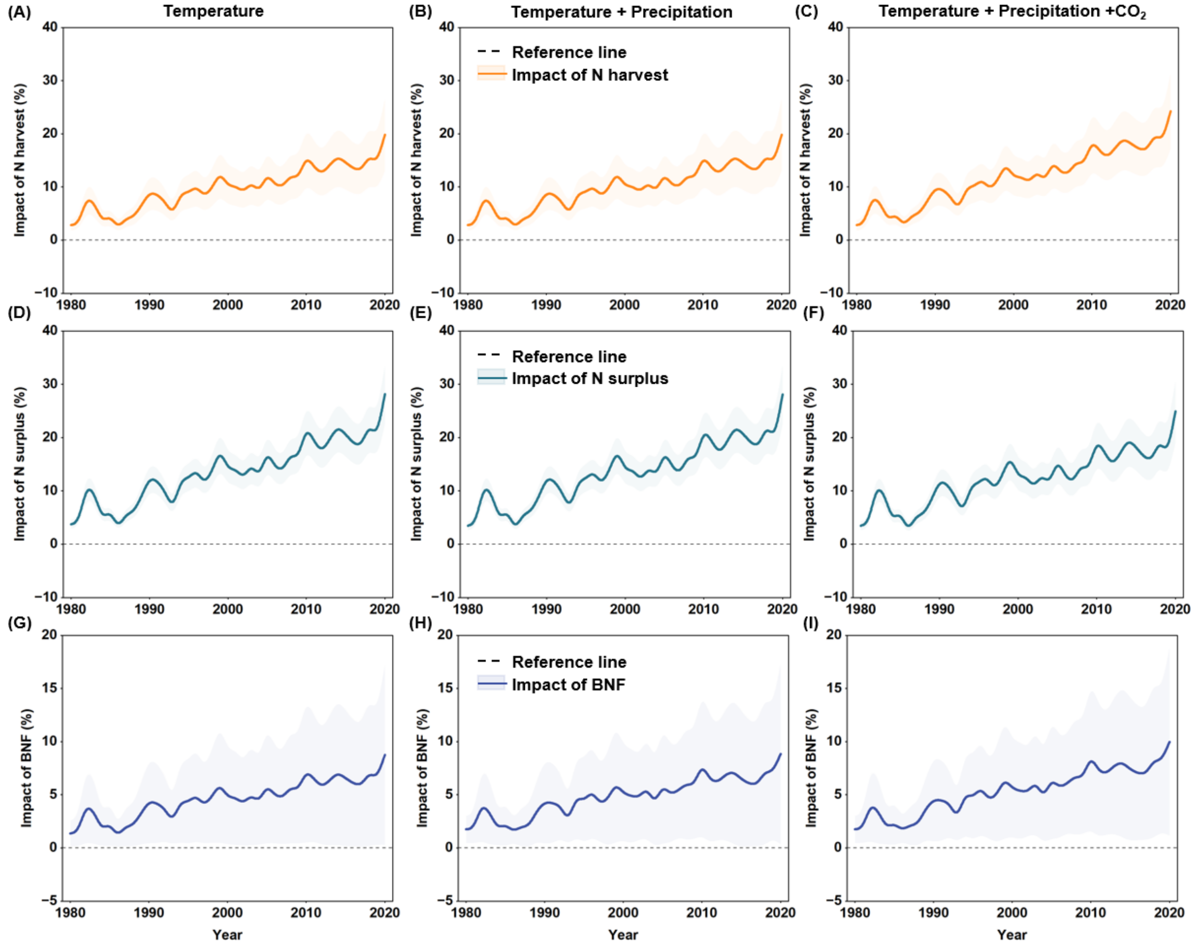

Fig. S2 Global impacts of climate changes on nitrogen harvest, nitrogen surplus, and biological nitrogen fixation. (A), (D), (G), Impacts of temperature change on nitrogen harvest **(A)**, nitrogen surplus **(D)**, and biological nitrogen fixation (BNF) **(G)** depicting their relative changes related to temperature change. **(B), (E), (H),** Impacts of temperature and precipitation changes on nitrogen harvest **(B)**, nitrogen surplus **(E)**, and BNF **(H)**. **(C), (F), (I),** Combined impacts of temperature, precipitation, and CO_2 changes on nitrogen harvest **(C)**, nitrogen surplus **(F)**, and BNF **(I)**. The black dashed line is the reference line indicating no impact and the shading represents a 90% confidence band. The solid line depicts the mean value of impact. In this figure, the global impact refers to the relative change (%). It is calculated by the impact divided by the actual observed value in the corresponding year, and the result is carried out in percentage terms.

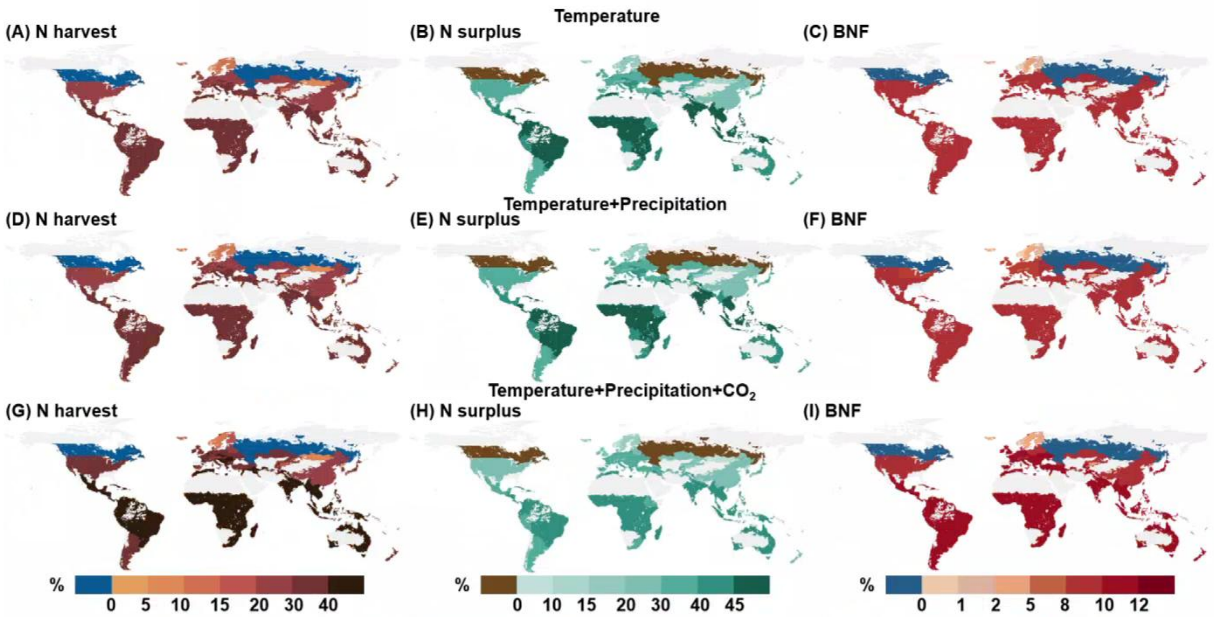

Fig. S3 Spatiotemporal variations of impacts of climate changes on nitrogen harvest, nitrogen surplus, and biological nitrogen fixation in 2020. Impacts of temperature changes on nitrogen harvest **(A)**, nitrogen surplus **(B)**, and biological nitrogen fixation (BNF) **(C)**. Impacts of temperature and precipitation changes on nitrogen harvest **(D)**, nitrogen surplus **(E)**, and BNF **(F)**. Combined impacts of temperature, precipitation, and CO_2 changes on nitrogen harvest **(G)**, nitrogen surplus **(H)**, and BNF **(I)**. The impact on each country represents the relative percentage change. It is determined by dividing the impact by the actual observed value in 2020, with the result expressed as a percentage. The base map is from GADM data, which are freely available for academic and other non-commercial use (https://gadm.org/).

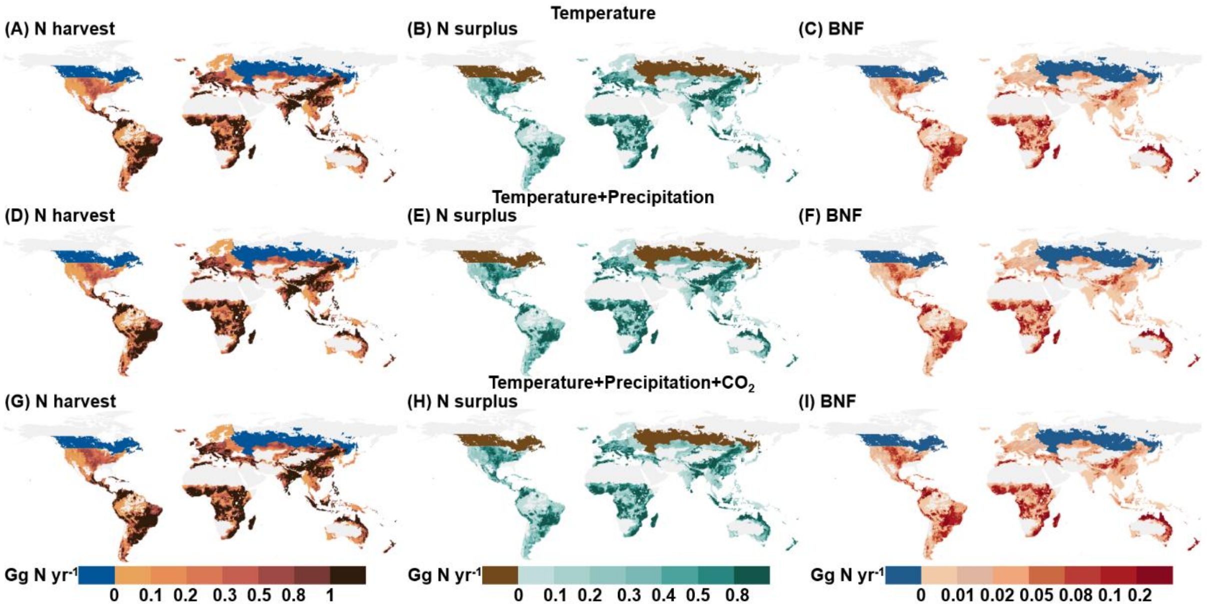

Fig. S4 Spatiotemporal variations of physical impacts of climate changes on nitrogen harvest, nitrogen surplus, and biological nitrogen fixation in 2020. Impacts of temperature changes on nitrogen harvest **(A)**, nitrogen surplus **(B)**, and biological nitrogen fixation (BNF) **(C)**. Impacts of temperature and precipitation changes on nitrogen harvest **(D)**, nitrogen surplus **(E)**, and BNF **(F)**. Combined impacts of temperature, precipitation, and CO_2 changes on nitrogen harvest **(G)**, nitrogen surplus **(H)**, and BNF **(I)**. The impact on each country represents the physical change compared to the actual observed value in 2020. Values in the legend demonstrate the average annual grassland N budget within a grid cell (0.5 by 0.5 degrees). The unit is Gg N yr^{-1}. The base map is from GADM data, which are freely available for academic and other non-commercial use (https://gadm.org/).

(A)

Scenario		Climate factor	Social-economic factor
Baseline scenario	SSP1	No climate change, fixed CO_2, temperature, precipitation levels since 2020	Sustainable society
	SSP2	No climate change, fixed CO_2, temperature, precipitation levels since 2020	BAU (middle road)
Climate change scenario	SSP1-2.6	Elevated CO_2, temperature, precipitation to RCP 2.6 level	Sustainable society
	SSP2-4.5	Elevated CO_2, temperature, precipitation to RCP 4.5 level	BAU (middle road)

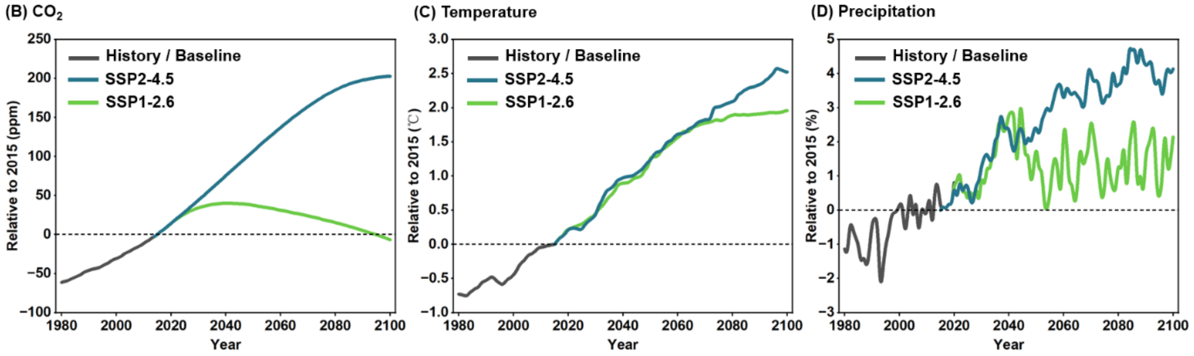

Fig. S5 Scenario design. (A) Concise narratives of the scenarios. **(B)** Historical and projected global atmosphere CO_2 changes from 1980-2100 compared to 2015 under the baseline and SSP-RCP scenarios. **(C)** Historical and projected global air temperature changes from 1980-2100 compared to 2015 under the baseline and SSP-RCP scenarios. **(D)** Historical and projected global land precipitation changes from 1980-2100 compared to 2015 under the baseline and SSP-RCP scenarios. SSP1-2.6, SSP1-RCP2.6; SSP2-4.5, SSP1-RCP4.5.

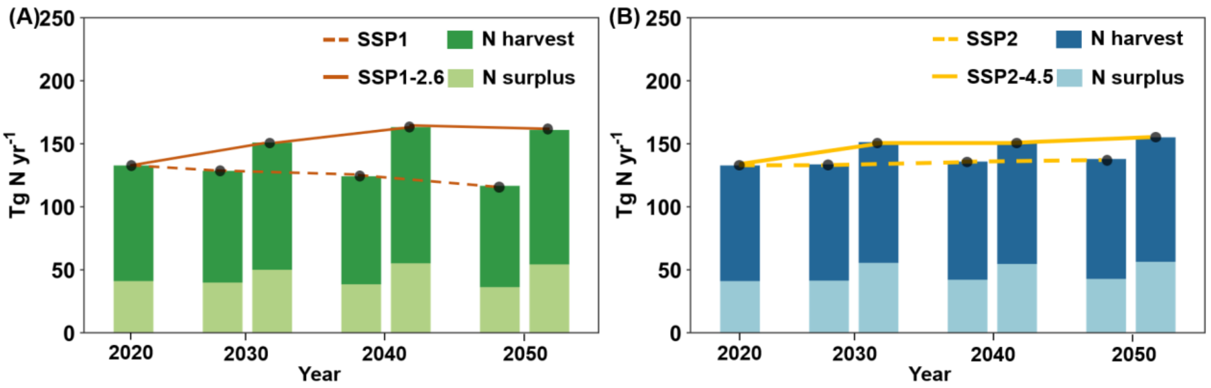

Fig. S6 Temporal variations in grassland N budgets under future scenarios from 2020 to 2050. (A) Variations under baseline SSP1 and climate change SSP1-2.6 scenarios; **(B)** Variations under baseline SSP2 and climate change SSP2-4.5 scenarios. Light green and light blue bars represent nitrogen surplus, while dark green and dark blue bars represent nitrogen harvest. The brown dashed line indicates nitrogen input under the baseline SSP1 scenario, and the brown solid line indicates nitrogen input under the climate change SSP1-2.6 scenario. Similarly, the yellow dashed line represents nitrogen input under the baseline SSP2 scenario, and the yellow solid line represents nitrogen input under the climate change SSP2-4.5 scenario.

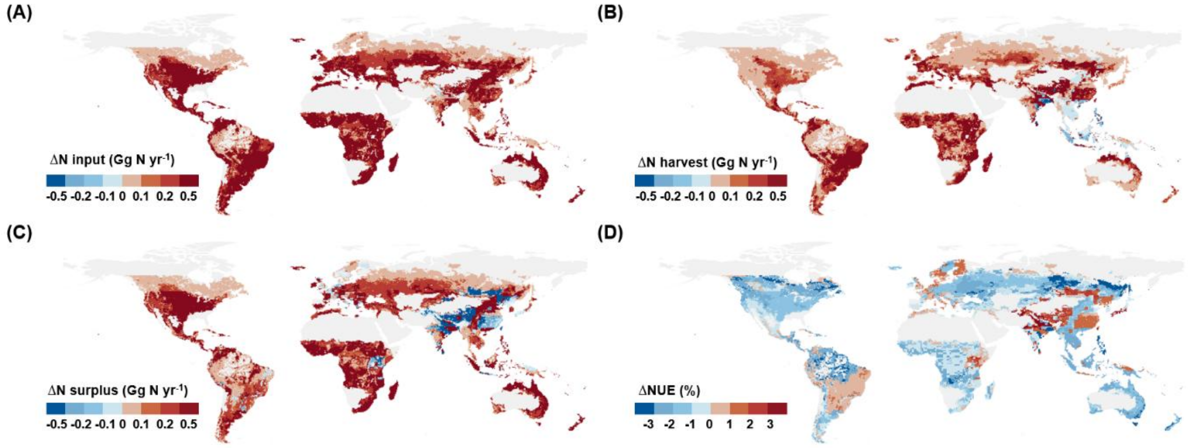

Fig. S7 Spatiotemporal variations of global grassland nitrogen budgets changes between baseline scenario and climate change SSP1-2.6 scenario in 2050. **(A)** ΔN input between baseline scenario and climate change SSP1-2.6 scenario in 2050; **(B)** ΔN harvest; **(C)** ΔN surplus; **(D)** ΔNUE. Values in the legend demonstrate the average annual grassland N budget within a grid cell (0.5 by 0.5 degrees). NUE, nitrogen use efficiency. The base map is from GADM data, which are freely available for academic and other non-commercial use (https://gadm.org/).

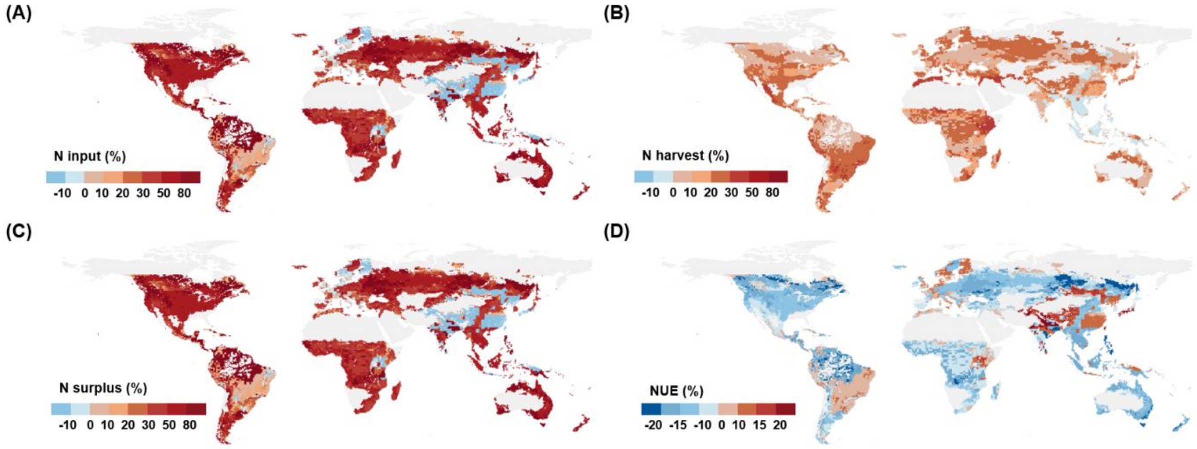

Fig. S8 Global grassland nitrogen budgets changes under the climate change SSP1-2.6 scenario in 2050. Value changes refer to the relative change (%) in this figure. It is calculated by the value difference simulated between baseline scenario and climate change SSP1-2.6 scenario in 2050 divided by the actual observed value in 2020 and the result is carried out in percentage terms. **(A)** N input; **(B)** N harvest; **(C)** N surplus; **(D)** NUE. Values in the legend demonstrate the average annual grassland N budget within a grid cell (0.5 by 0.5 degrees). NUE, nitrogen use efficiency. The base map is from GADM data, which are freely available for academic and other non-commercial use (https://gadm.org/).

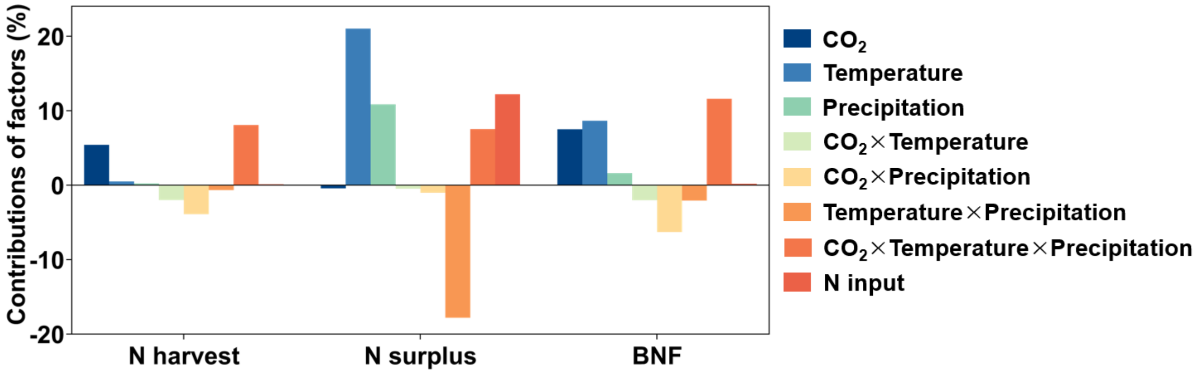

Fig. S9 Contributions of individual climatic factors to total climate impacts under the SSP1-2.6 scenario by 2050. CO_2, temperature and precipitation are the mean carbon dioxide concentrations (10^3ppm), mean annual temperature (10^2°C), and mean annual precipitation (10^3 mm), respectively; CO_2×Temperature, the interaction between CO_2 and temperature (10^4 ppm*°C); CO_2×Precipitation, the interaction between CO_2 and precipitation (10^5 ppm*mm); Temperature×Precipitation, the interaction between temperature and precipitation (10^3 °C*mm); CO_2×Temperature×Precipitation, the interaction among CO_2, temperature, and precipitation (10^6 ppm*°C*mm). BNF, biological nitrogen fixation.

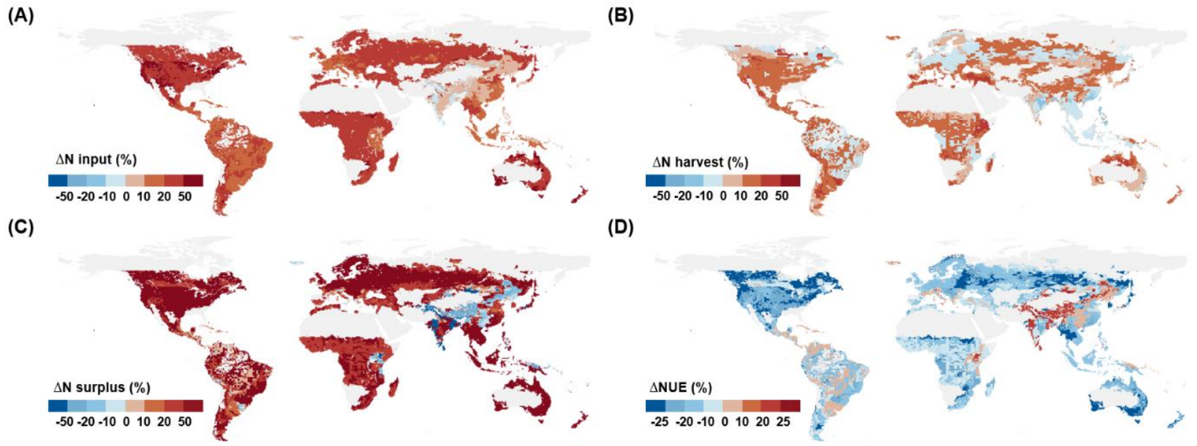

Fig. S10 Global grassland nitrogen budgets changes under the climate change SSP2-4.5 scenario in 2050. Value changes refer to the relative change (%) in this figure. It is calculated by the value difference simulated between baseline scenario and climate change SSP2-4.5 scenario in 2050 divided by the actual observed value in 2020 and the result is carried out in percentage terms. **(A)** N input; **(B)** N harvest; **(C)** N surplus; **(D)** NUE. Values in the legend demonstrate the average annual grassland N budget within a grid cell (0.5 by 0.5 degrees). NUE, nitrogen use efficiency. The base map is from GADM data, which are freely available for academic and other non-commercial use (https://gadm.org/).

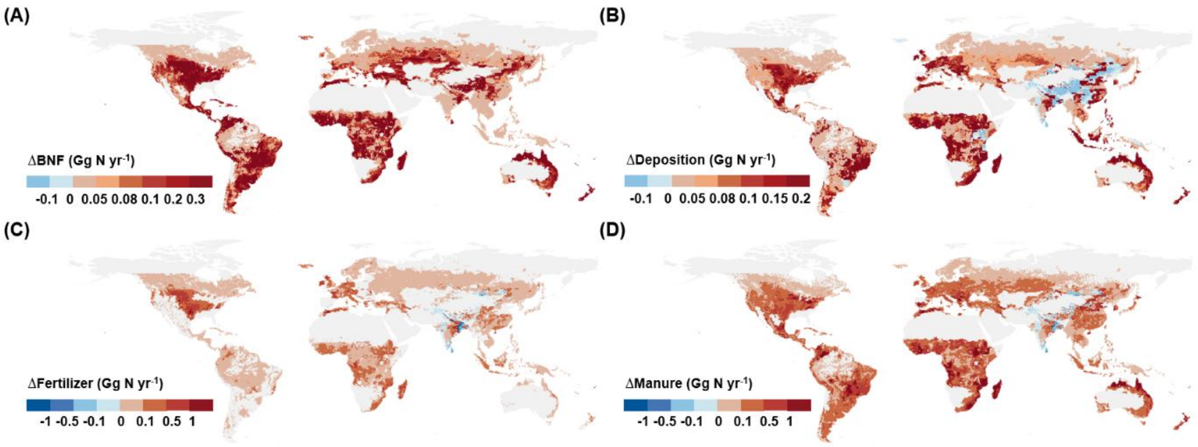

Fig. S11 Spatiotemporal variations of global grassland nitrogen input budgets changes between baseline scenario and climate change SSP2-4.5 scenario in 2050. **(A)** ΔBNF between baseline scenario and climate change SSP2-4.5 scenario in 2050; **(B)** ΔDeposition; **(C)** ΔFertilizer; **(D)** ΔManure. Values in the legend demonstrate the average annual grassland N budget within a grid cell (0.5 by 0.5 degrees). The unit is Gg N yr^{-1}. BNF, biological nitrogen fixation. The base map is from GADM data, which are freely available for academic and other non-commercial use (https://gadm.org/).

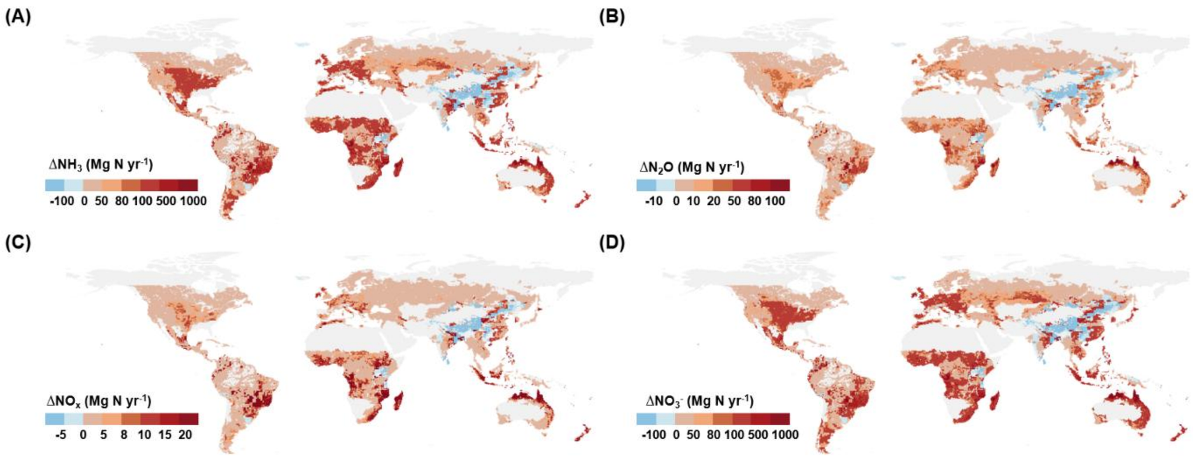

Fig. S12 Spatiotemporal variations of global grassland nitrogen surplus budgets changes between baseline scenario and climate change SSP2-4.5 scenario in 2050. (A) ΔNH_3 between baseline scenario and climate change SSP2-4.5 scenario in 2050; **(B)** ΔN_2O; **(C)** ΔNO_x; **(D)** ΔNO_3^-. Values in the legend demonstrate the average annual grassland N budget within a grid cell (0.5 by 0.5 degrees). The unit is Mg N yr^{-1}. The base map is from GADM data, which are freely available for academic and other non-commercial use (https://gadm.org/).

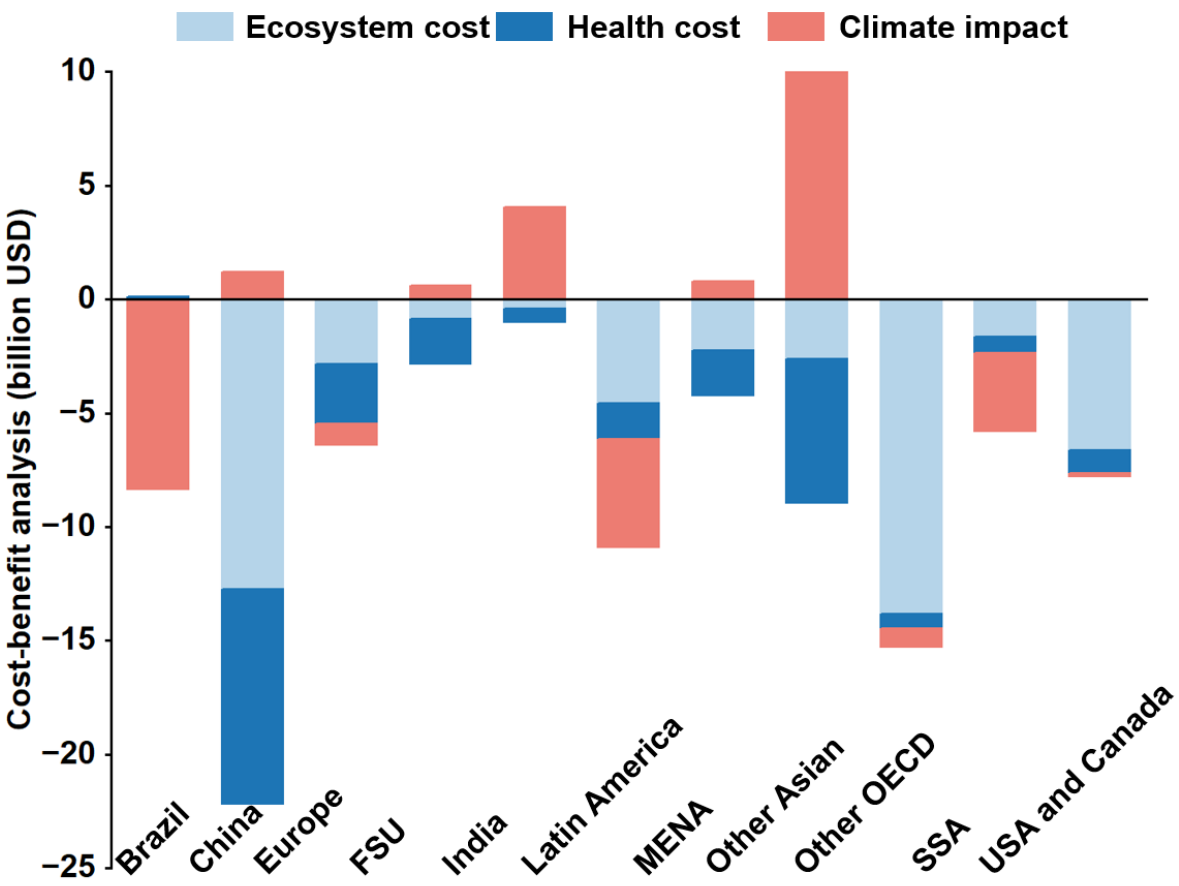

Fig. S13 Cost-benefit analysis of climate changes in global grasslands under the by 2050. This analysis compares the cost-benefit outcomes of the climate change SSP2-4.5 scenario against the baseline scenario for global grasslands by 2050. Positive values indicate benefits, while negative values represent costs. FSU, Former Soviet Union; MENA, Middle East and North Africa; OECD, Organization for Economic Cooperation and Development; SSA, Sub-Saharan Africa.

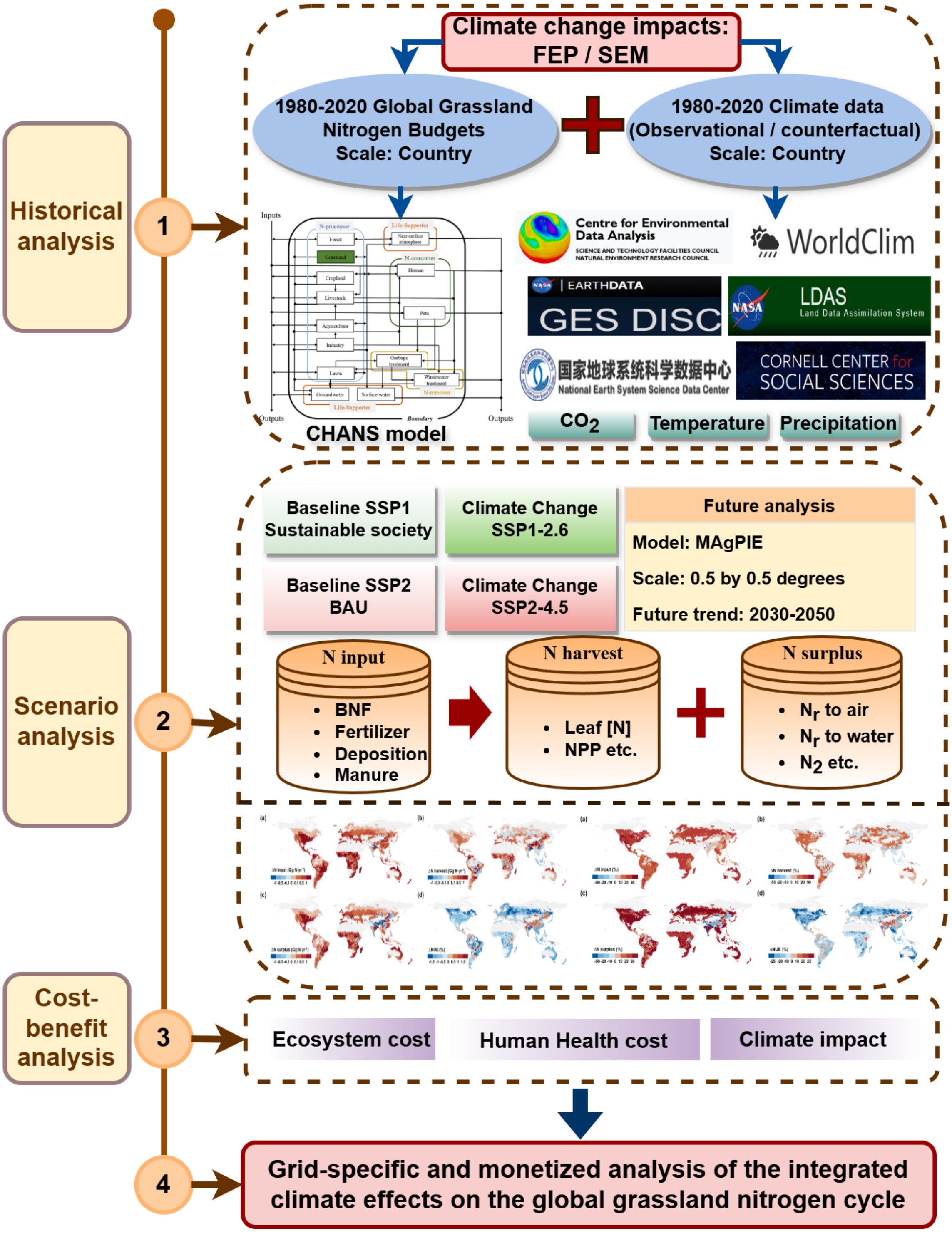

Fig. S14 Methodology framework. FEP, fixed effect panel model; SEM, structural equation model; CHANS, Coupled Human and Natural Systems; MAgPIE, The Model of Agricultural Production and its Impact on the Environment; BNF, biological N fixation; NPP, net primary productivity; Leaf [N], leaf N content; N_r, reactive N.

Table S1 Effects of climate change on nitrogen harvest, nitrogen surplus, and biological nitrogen fixation changes.

	Ln N harvest (Tg)		Ln N surplus (Tg)		Ln BNF (Tg)	
	Coefficient	Effects	Coefficient	Effects	Coefficient	Effects
CO_2 (10^3 ppm)	0.043	1.771%	-0.030	-1.257%	0.011	0.406%
Temperature (10^2 °C)	0.409	16.854%	0.488	20.453%	0.492	18.162%
Temperature^2 (10^2 °C)	-0.126	-5.189%	-0.101	-4.233%	-0.339	-12.514%
Precipitation (10^3 mm)	0.885	36.450%	0.895	37.510%	0.330	12.182%
Precipitation^2 (10^3 mm)	-0.006	-0.247%	0.016	0.671%	-0.022	-0.812%
CO_2× Temperature (10^4 ppm×°C)	-0.352	-14.498%	-0.423	-17.728%	-0.350	-12.920%
CO_2×Precipitation (10^5 ppm×mm)	-0.927	-38.180%	-0.935	-39.187%	-0.113	-4.171%
Temperature×Precipitation (10^3 °C×mm)	-1.382	-56.919%	-1.449	-60.729%	-0.298	-11.000%
CO_2×Temperature×Precipitation (10^6 ppm×°C×mm)	1.464	60.297%	1.479	61.987%	0.405	14.950%
Ln BNF (Tg)	0.271	11.161%	0.224	9.388%		
Ln Fertilizer (Tg)	0.160	6.590%	0.164	6.873%	0.003	0.111%
Ln Manure (Tg)	0.289	11.903%	0.303	12.699%	-0.018	-0.664%

Note: The coefficient refers to standardization coefficient based on models in Table 1. The effects of each item were derived from the ratio of the standardization coefficient of each explanatory variable to the standard deviation of the explained variable. BNF, biological nitrogen fixation.

Table S2 Summary of the structural equation model of climate changes on nitrogen harvest.

Driving factors	CO_2 (10^1ppm)		Temperature (10^1°C)		Precipitation (10^2 mm)		Evapotranspiration (10^2 mm)	
	Effect	Ratio	Effect	Ratio	Effect	Ratio	Effect	Ratio
**Direct effect**	0.17	59%	0.24	44%	0.08	-17%	0.01	6%
**Indirect effect from soil C:N**	0.03	10%	-0.03	-5%	0.01	-2%	0.01	6%
**Indirect effect from BNF**	0.09	31%	0.33	61%	-0.56	119%	0.16	88%
**Net effect**	0.29		0.54		-0.47		0.18	

Notes: Direct effects are derived from the standardized path coefficient from the variables to N harvest based on Figure 2. Indirect effects were calculated as the product of all effects in a single path. Net effects are the sum of all direct and indirect effects. Ratio is the value of direct or indirect effects divided by the values of net effects. Soil C:N ratio, soil carbon content ratio divided by nitrogen content ratio; BNF, biological nitrogen fixation.

Table S3 Summary of the structural equation model of climate changes on nitrogen surplus.

Driving factors	CO_2 (10^1ppm)		Temperature (10^1°C)		Precipitation (10^2 mm)		Evapotranspiration (10^2 mm)	
	Effect	Ratio	Effect	Ratio	Effect	Ratio	Effect	Ratio
**Direct effect**	-0.37	148%	0.07	19%	0.02	-4%	0.04	19%
**Indirect effect from soil C:N**	0.03	-12%	-0.02	-5%	0.01	-2%	0.01	5%
**Indirect effect from BNF**	0.09	-36%	0.32	86%	-0.56	106%	0.16	76%
**Net effect**	-0.25		0.37		-0.53		0.21	

Notes: Direct effects are derived from the standardized path coefficient from the variables to N surplus based on Figure 2. Indirect effects were calculated as the product of all effects in a single path. Net effects are the sum of all direct and indirect effects. Ratio is the value of direct or indirect effects divided by the values of net effects. Soil C:N ratio, soil carbon content ratio divided by nitrogen content ratio; BNF, biological nitrogen fixation.

Table S4 Nitrogen fluxes in global grasslands under the baseline scenario and climate change SSP2-4.5 scenario by 2050.

Variable	Scenario value	Component	Scenario value
**N input (Tg)**	Baseline：138.0<br>Climate change：160.3	BNF (Tg)	Baseline：15.8<br>Climate change：22.2
		Deposition (Tg)	Baseline：15.6<br>Climate change：18.9
		Fertilizer (Tg)	Baseline：26.8<br>Climate change：32.7
		Manure (Tg)	Baseline：79.8<br>Climate change：86.5
**N harvest (Tg)**	Baseline：95.1<br>Climate change：102.3		
**N surplus (Tg)**	Baseline：42.9<br>Climate change：58.0	NH_3 (Tg)	Baseline：9.2<br>Climate change：12.4
		N_2O (Tg)	Baseline：1.0<br>Climate change：1.3
		NO_x (Tg)	Baseline：0.4<br>Climate change：0.5
		NO_3^- (Tg)	Baseline：10.8<br>Climate change：14.6
		N_2 (Tg)	Baseline：21.5<br>Climate change：29.2
**NUE (%)**	Baseline：68.9<br>Climate change：63.8		

Note: BNF, biological N fixation; NUE, nitrogen use efficiency.

Table S5 Residual variation in weather variables.

	CO_2 (10^3 ppm)		Temperature (10^2 °C)		Precipitation (10^3mm)	
	R^2	SD of Residual	R^2	SD of Residual	R^2	SD of Residual
**No FE**		0.0214		0.0764		0.0557
**Year FE**	0.991	0.0020	0.002	0.0763	0.003	0.0556
**Country FE**	0.006	0.0213	0.995	0.0055	0.951	0.0123
**Year FE+ Country FE**	0.997	0.0013	0.997	0.0042	0.954	0.0120

Notes: Variations in CO_2, temperature, and precipitation are absorbed by fixed effects (FE). This table presents the regression results of these weather variables under different fixed effect specifications. The SD of Residual represents the standard deviation of the residuals, indicating the remaining variation in the weather variables.

Table S6 Summary statistics for regression analysis.

	Obs	Mean	SD	Min	Max
Ln N harvest (Tg)	6,647	-3.73	2.93	-16.76	1.75
Ln N surplus (Tg)	6,666	-3.87	2.87	-15.15	1.45
Ln BNF (Tg)	6,458	-3.85	2.92	-14.69	1.78
Ln Fertilizer (Tg)	3,928	-6.52	3.99	-42.98	0.49
Ln Manure (Tg)	6,581	-4.68	2.29	-14.34	0.03
Temperature (10^2 °C)	6,806	0.18	0.08	-0.01	0.31
Precipitation (10^3 mm)	6,806	0.09	0.06	0.00	0.33
CO_2 (10^3 ppm)	6,806	0.37	0.02	0.34	0.42
Grassland area (10^7 ha)	6,806	1.86	5.42	0.00	46.28
Manure-fertilizer ratio (10^{12})	4,113	0.18	11.7	-0.00	747
Biological fixation ratio (10^{12})	4,113	0.06	3.69	-0.00	237

Note: BNF, biological N fixation.

Table S7 Robust tests of regression of nitrogen harvest, nitrogen surplus, biological nitrogen fixation, fertilizer, and manure with the quadratic term of CO_2.

	Ln N harvest (Tg)	Ln N surplus (Tg)	Ln BNF (Tg)	Ln Fertilizer (Tg)	Ln Manure (Tg)
	Model 1	Model 2	Model 3	Model 4	Model 5
CO_2 (10^3 ppm)	2.906***	-2.810	-1.352	-19.928***	-2.469***
s.e.	(0.481)	(0.391)	(0.609)	(2.055)	(0.457)
CO_2^2 (10^3 ppm)	-1.241	-4.218	-0.154	-25.696	-3.243
s.e.	(0.613)	(0.498)	(0.773)	(2.615)	(0.585)
Temperature (10^2 °C)	13.277***	15.365***	16.448***	-50.575***	-31.587***
s.e.	(2.177)	(1.767)	(2.738)	(9.509)	(1.913)
$Temperature^2$ (10^2 °C)	-8.152*	-5.832**	-34.924***	-24.381	20.810***
s.e.	(5.671)	(4.600)	(7.140)	(25.051)	(5.017)
Precipitation (10^3 mm)	36.895***	36.702***	2.636	-6.386	12.774**
s.e.	(5.089)	(4.129)	(6.431)	(22.526)	(5.069)
$Precipitation^2$ (10^3 mm)	-1.160	2.471	-4.043	-30.745**	4.705*
s.e.	(2.713)	(2.199)	(3.425)	(12.025)	(2.547)
CO_2 × Temperature (10^4 ppm × °C)	-3.121***	-3.590***	-2.969***	13.131***	6.775***
s.e.	(0.588)	(0.477)	(0.742)	(2.588)	(0.489)
CO_2 × Precipitation (10^5 ppm × mm)	-10.036***	-9.958***	-1.409	0.895	-3.954***
s.e.	(1.328)	(1.078)	(1.678)	(5.879)	(1.328)
Temperature × Precipitation (10^3 °C × mm)	-2.011***	-2.073***	-0.486*	2.984***	-0.263
s.e.	(0.223)	(0.181)	(0.281)	(0.982)	(0.211)
CO_2 × Temperature × Precipitation (10^6 ppm × °C × mm)	5.541***	5.500***	1.713**	-6.235**	0.853
s.e.	(0.573)	(0.465)	(0.724)	(2.527)	(0.551)
Ln BNF (Tg)	0.243***	0.197***			
s.e.	(0.013)	(0.011)			
Ln Fertilizer (Tg)	0.098***	0.098***	0.002		
s.e.	(0.004)	(0.003)	(0.005)		
Ln Manure (Tg)	0.327***	0.338***	-0.024		
s.e.	(0.017)	(0.014)	(0.022)		

Country	Yes	Yes	Yes	Yes	Yes
Year	Yes	Yes	Yes	Yes	Yes
N	3,811	3,817	3,817	3,928	5,581
Adj-R^2	0.992	0.994	0.989	0.942	0.986

Notes: each column representing a separate regression model, i.e., the fixed effect panel (FEP) model. Model 1, 2, 3, 4, and 5 present the regression results responding to nitrogen harvest, nitrogen surplus, biological nitrogen fixation (BNF), fertilizer and manure, respectively. The quadratic item of CO_2 was further added in Model 1-5. Significance levels based on p-values are indicated by asterisks: *P < 0.1, **P < 0.05, ***P < 0.01. Standard errors are shown in parentheses. N, the number of samples. Each regression equation includes control variables: grassland area, ratio of organic and synthetic fertilizers, and ratio of biological nitrogen fixation. All equations include country and year fixed effects.

Supplementary Data

Global and country-level grassland nitrogen budget (1980–2020) from the CHANS model, including both managed and undisturbed grasslands.

REFERENCES

1. Intergovernmental Panel on Climate Change (IPCC), *Climate Change 2021 – The Physical Science Basis: Working Group I Contribution to the Sixth Assessment Report of the Intergovernmental Panel on Climate Change* (Cambridge Univ. Press, ed. 1, 2023).
2. L. Xia, S. K. Lam, R. Kiese, D. Chen, Y. Luo, K. J. Van Groenigen, E. A. Ainsworth, J. Chen, S. Liu, L. Ma, Y. Zhu, K. Butterbach-Bahl, Elevated CO_2 negates O_3 impacts on terrestrial carbon and nitrogen cycles. *One Earth* **4**, 1752–1763 (2021).
3. C. W. Thackeray, A. Hall, J. Norris, D. Chen, Constraining the increased frequency of global precipitation extremes under warming. *Nat. Clim. Change* **12**, 441–448 (2022).
4. J. Zhao, T. Y. Gan, G. Zhang, S. Zhang, Projected changes of precipitation extremes in North America using CMIP6 multi-climate model ensembles. *J. Hydrol.* **621**, 129598 (2023).
5. Y. Bai, M. F. Cotrufo, Grassland soil carbon sequestration: Current understanding, challenges, and solutions. *Science* **377**, 603–608 (2022).
6. E. Lugato, J. M. Lavallee, M. L. Haddix, P. Panagos, M. F. Cotrufo, Different climate sensitivity of particulate and mineral-associated soil organic matter. *Nat. Geosci.* **14**, 295–300 (2021).
7. Z. Xu, H. Shimizu, S. Ito, Y. Yagasaki, C. Zou, G. Zhou, Y. Zheng, Effects of elevated CO_2, warming and precipitation change on plant growth, photosynthesis and peroxidation in dominant species from North China grassland. *Planta* **239**, 421–435 (2014).
8. X. Li, J. A. Palta, F. Liu, Editorial: Modulation of stomatal response by elevated CO_2 in plants under drought and heat stress. *Front. Plant Sci.* **13**, 843999 (2022).
9. L. Lin, B. Zhu, C. Chen, Z. Zhang, Q.-B. Wang, J.-S. He, Precipitation overrides warming in mediating soil nitrogen pools in an alpine grassland ecosystem on the Tibetan Plateau. *Sci. Rep.* **6**, 31438 (2016).
10. L. D. Simba, M. Te Beest, H.-J. Hawkins, K. W. Larson, A. R. Palmer, C. Sandström, K. G. Smart, G. I. H. Kerley, J. P. G. M. Cromsigt, Wilder rangelands as a natural climate opportunity: Linking climate action to biodiversity conservation and social transformation. *Ambio* **53**, 678–696 (2024).
11. E. Bai, S. Li, W. Xu, W. Li, W. Dai, P. Jiang, A meta-analysis of experimental warming effects on terrestrial nitrogen pools and dynamics. *New Phytol.* **199**, 441–451 (2013).
12. P. M. Homyak, S. D. Allison, T. E. Huxman, M. L. Goulden, K. K. Treseder, Effects of drought manipulation on soil nitrogen cycling: A meta-analysis. *J. Geophys. Res. Biogeosci.* **122**, 3260–

3272 (2017).

13. J. Song, S. Wan, S. Piao, A. K. Knapp, A. T. Classen, S. Vicca, P. Ciais, M. J. Hovenden, S. Leuzinger, C. Beier, P. Kardol, J. Xia, Q. Liu, J. Ru, Z. Zhou, Y. Luo, D. Guo, J. Adam Langley, J. Zscheischler, J. S. Dukes, J. Tang, J. Chen, K. S. Hofmockel, L. M. Kueppers, L. Rustad, L. Liu, M. D. Smith, P. H. Templer, R. Quinn Thomas, R. J. Norby, R. P. Phillips, S. Niu, S. Fatichi, Y. Wang, P. Shao, H. Han, D. Wang, L. Lei, J. Wang, X. Li, Q. Zhang, X. Li, F. Su, B. Liu, F. Yang, G. Ma, G. Li, Y. Liu, Y. Liu, Z. Yang, K. Zhang, Y. Miao, M. Hu, C. Yan, A. Zhang, M. Zhong, Y. Hui, Y. Li, M. Zheng, A meta-analysis of 1,119 manipulative experiments on terrestrial carbon-cycling responses to global change. *Nat. Ecol. Evol.* **3**, 1309–1320 (2019).
14. N. Wang, B. Quesada, L. Xia, K. Butterbach-Bahl, C. L. Goodale, R. Kiese, Effects of climate warming on carbon fluxes in grasslands—A global meta-analysis. *Glob. Change Biol.* **25**, 1839–1851 (2019).
15. Z. Zhou, C. Wang, Y. Luo, Meta-analysis of the impacts of global change factors on soil microbial diversity and functionality. *Nat. Commun.* **11**, 3072 (2020).
16. Y. Sun, C. Wang, H. Y. H. Chen, Q. Liu, B. Ge, B. Tang, A global meta-analysis on the responses of C and N concentrations to warming in terrestrial ecosystems. *Catena* **208**, 105762 (2022).
17. M. Zheng, J. Cui, L. Cheng, X. Wang, X. Zhang, S. K. Lam, B. Gu, Warming promotes nitrogen and carbon cycles in global grassland. *Environ. Sci. Technol.* **59**, 2505–2518 (2025).
18. H. Liu, Z. Mi, L. Lin, Y. Wang, Z. Zhang, F. Zhang, H. Wang, L. Liu, B. Zhu, G. Cao, X. Zhao, N. J. Sanders, A. T. Classen, P. B. Reich, J.-S. He, Shifting plant species composition in response to climate change stabilizes grassland primary production. *Proc. Natl. Acad. Sci. U.S.A.* **115**, 4051–4056 (2018).
19. X. Xin, D. Jin, Y. Ge, J. Wang, J. Chen, J. Qi, H. Chu, C. Shao, P. J. Murray, R. Zhao, Q. Qin, H. Tang, Climate change dominated long-term soil carbon losses of Inner Mongolian grasslands. *Global Biogeochem. Cycles* **34**, e2020GB006559 (2020).
20. J. P. Dietrich, B. L. Bodirsky, F. Humpenöder, I. Weindl, M. Stevanović, K. Karstens, U. Kreidenweis, X. Wang, A. Mishra, D. Klein, G. Ambrósio, E. Araujo, A. W. Yalew, L. Baumstark, S. Wirth, A. Giannousakis, F. Beier, D. M.-C. Chen, H. Lotze-Campen, A. Popp, MAgPIE 4—A modular open-source framework for modeling global land systems. *Geosci. Model Dev.* **12**, 1299–1317 (2019).

21. B. Gu, X. Ju, J. Chang, Y. Ge, P. M. Vitousek, Integrated reactive nitrogen budgets and future trends in China. *Proc. Natl. Acad. Sci. U.S.A.* **112**, 8792–8797 (2015).
22. B. Gu, Y. Ge, Y. Ren, B. Xu, W. Luo, H. Jiang, B. Gu, J. Chang, Atmospheric reactive nitrogen in China: Sources, recent trends, and damage costs. *Environ. Sci. Technol.* **46**, 9420–9427 (2012).
23. B. Gu, S. K. Lam, S. Reis, H. Van Grinsven, X. Ju, X. Yan, F. Zhou, H. Liu, Z. Cai, J. N. Galloway, C. Howard, M. A. Sutton, D. Chen, Toward a generic analytical framework for sustainable nitrogen management: Application for China. *Environ. Sci. Technol.* **53**, 1109–1118 (2019).
24. B. Gu, X. Zhang, S. K. Lam, Y. Yu, H. J. M. Van Grinsven, S. Zhang, X. Wang, B. L. Bodirsky, S. Wang, J. Duan, C. Ren, L. Bouwman, W. De Vries, J. Xu, M. A. Sutton, D. Chen, Cost-effective mitigation of nitrogen pollution from global croplands. *Nature* **613**, 77–84 (2023).
25. J. M. Craine, J. B. Nippert, A. J. Elmore, A. M. Skibbe, S. L. Hutchinson, N. A. Brunsell, Timing of climate variability and grassland productivity. *Proc. Natl. Acad. Sci. U.S.A.* **109**, 3401–3405 (2012).
26. N. El Haddad, H. Choukri, M. E. Ghanem, A. Smouni, R. Mentag, K. Rajendran, K. Hejjaoui, F. Maalouf, S. Kumar, High-temperature and drought stress effects on growth, yield and nutritional quality with transpiration response to vapor pressure deficit in Lentil. *Plants* **11**, 95 (2021).
27. F. N. Tubiello, J.-F. Soussana, S. M. Howden, Crop and pasture response to climate change. *Proc. Natl. Acad. Sci. U.S.A.* **104**, 19686–19690 (2007).
28. J. A. Morgan, "Rising atmospheric CO_2 and global climate change: Responses and management implications for grazing lands," in *Grasslands* (CRC Press, 2019), pp. 235–260.
29. M. Reyes-Fox, H. Steltzer, M. J. Trlica, G. S. McMaster, A. A. Andales, D. R. LeCain, J. A. Morgan, Elevated CO_2 further lengthens growing season under warming conditions. *Nature* **510**, 259–262 (2014).
30. K. Hufkens, T. F. Keenan, L. B. Flanagan, R. L. Scott, C. J. Bernacchi, E. Joo, N. A. Brunsell, J. Verfaillie, A. D. Richardson, Productivity of North American grasslands is increased under future climate scenarios despite rising aridity. *Nat. Clim. Change* **6**, 710–714 (2016).
31. M. R. Shaw, E. S. Zavaleta, N. R. Chiariello, E. E. Cleland, H. A. Mooney, C. B. Field, Grassland responses to global environmental changes suppressed by elevated CO_2. *Science* **298**,

1987–1990 (2002).

32. R. A. Gill, H. W. Polley, H. B. Johnson, L. J. Anderson, H. Maherali, R. B. Jackson, Nonlinear grassland responses to past and future atmospheric CO_2. *Nature* **417**, 279–282 (2002).
33. G. Luo, C. Xue, Q. Jiang, Y. Xiao, F. Zhang, S. Guo, Q. Shen, N. Ling, Soil carbon, nitrogen, and phosphorus cycling microbial populations and their resistance to global change depend on soil C:N:P stoichiometry. *mSystems* **5**, 10.1128/msystems.00162-20 (2020).
34. P. Duan, D. Zhao, S. Yang, J. Chen, Z. Chen, L. Cao, Soil native C/N ratio affects diazotrophic bacterial composition and N fixation by regulating SOC distribution in soil particles after residue incorporation. *Microorganisms* **13**, 1104 (2025).
35. Z. Dai, M. Yu, H. Chen, H. Zhao, Y. Huang, W. Su, F. Xia, S. X. Chang, P. C. Brookes, R. A. Dahlgren, J. Xu, Elevated temperature shifts soil N cycling from microbial immobilization to enhanced mineralization, nitrification and denitrification across global terrestrial ecosystems. *Glob. Change Biol.* **26**, 5267–5276 (2020).
36. J. Dong, N. Gruda, X. Li, Y. Tang, Z. Duan, Impacts of elevated CO_2 on nitrogen uptake of cucumber plants and nitrogen cycling in a greenhouse soil. *Appl. Soil Ecol.* **145**, 103342 (2020).
37. E. S. Zavaleta, M. R. Shaw, N. R. Chiariello, H. A. Mooney, C. B. Field, Additive effects of simulated climate changes, elevated CO_2, and nitrogen deposition on grassland diversity. *Proc. Natl. Acad. Sci. U.S.A.* **100**, 7650–7654 (2003).
38. J. Sun, Y. Wang, S. Piao, M. Liu, G. Han, J. Li, E. Liang, T. M. Lee, G. Liu, A. Wilkes, S. Liu, W. Zhao, H. Zhou, M. Yibeltal, M. L. Berihun, D. Browning, A. A. Fenta, A. Tsunekawa, J. Brown, W. Willms, M. Tsubo, Toward a sustainable grassland ecosystem worldwide. *Innovation* **3**, 100265 (2022).
39. B. L. Bodirsky, A. Popp, H. Lotze-Campen, J. P. Dietrich, S. Rolinski, I. Weindl, C. Schmitz, C. Müller, M. Bonsch, F. Humpenöder, A. Biewald, M. Stevanovic, Reactive nitrogen requirements to feed the world in 2050 and potential to mitigate nitrogen pollution. *Nat. Commun.* **5**, 3858 (2014).
40. A. Popp, K. Calvin, S. Fujimori, P. Havlik, F. Humpenöder, E. Stehfest, B. L. Bodirsky, J. P. Dietrich, J. C. Doelmann, M. Gusti, T. Hasegawa, P. Kyle, M. Obersteiner, A. Tabeau, K. Takahashi, H. Valin, S. Waldhoff, I. Weindl, M. Wise, E. Kriegler, H. Lotze-Campen, O. Fricko, K. Riahi, D. P. V. Vuuren, Land-use futures in the shared socio-economic pathways. *Glob. Environ. Change* **42**, 331–345 (2017).

41. Y. Hautier, E. W. Seabloom, E. T. Borer, P. B. Adler, W. S. Harpole, H. Hillebrand, E. M. Lind, A. S. MacDougall, C. J. Stevens, J. D. Bakker, Y. M. Buckley, C. Chu, S. L. Collins, P. Daleo, E. I. Damschen, K. F. Davies, P. A. Fay, J. Firn, D. S. Gruner, V. L. Jin, J. A. Klein, J. M. H. Knops, K. J. La Pierre, W. Li, R. L. McCulley, B. A. Melbourne, J. L. Moore, L. R. O'Halloran, S. M. Prober, A. C. Risch, M. Sankaran, M. Schuetz, A. Hector, Eutrophication weakens stabilizing effects of diversity in natural grasslands. *Nature* **508**, 521–525 (2014).
42. R. W. Kates, W. R. Travis, T. J. Wilbanks, Transformational adaptation when incremental adaptations to climate change are insufficient. *Proc. Natl. Acad. Sci. U.S.A.* **109**, 7156–7161 (2012).
43. A. Chaturvedi, B. Pandey, A. K. Yadav, S. Saroj, An overview of the potential impacts of global climate change on water resources. *Water Conserv. Era Glob. Clim. Change* 99–120 (2021).
44. L. B. Kimmell, J. M. Fagan, C. A. Havrilla, Soil restoration increases soil health across global drylands: A meta-analysis. *J. Appl. Ecol.* **60**, 1939–1951 (2023).
45. D. Tilman, C. Balzer, J. Hill, B. L. Befort, Global food demand and the sustainable intensification of agriculture. *Proc. Natl. Acad. Sci. U.S.A.* **108**, 20260–20264 (2011).
46. B. C. O'Neill, L. Jiang, S. Kc, R. Fuchs, S. Pachauri, E. K. Laidlaw, T. Zhang, W. Zhou, X. Ren, The effect of education on determinants of climate change risks. *Nat. Sustain.* **3**, 520–528 (2020).
47. A. Mayer, Z. Hausfather, A. D. Jones, W. L. Silver, The potential of agricultural land management to contribute to lower global surface temperatures. *Sci. Adv.* **4**, eaaq0932 (2018).
48. S. Roe, C. Streck, M. Obersteiner, S. Frank, B. Griscom, L. Drouet, O. Fricko, M. Gusti, N. Harris, T. Hasegawa, Z. Hausfather, P. Havlík, J. House, G.-J. Nabuurs, A. Popp, M. J. S. Sánchez, J. Sanderman, P. Smith, E. Stehfest, D. Lawrence, Contribution of the land sector to a 1.5°C world. *Nat. Clim. Change* **9**, 817–828 (2019).
49. J. G. Oliveira, M. L. S. Júnior, N. J. C. Maia, J. C. B. D. Junior, A. H. Gameiro, T. R. Kunrath, G. G. Mendonça, F. F. Simili, Nitrogen balance and efficiency as indicators for monitoring the proper use of fertilizers in agricultural and livestock systems. *Sci. Rep.* **12**, 12021 (2022).
50. IFA, "Fertilizer best management practices: General principles, strategy for their adoption and voluntary initiatives vs regulations," in *International Workshop on Best Fertilizer Management Practices* (International Fertilizer Industry Association, 2007).

51. N. M. Capstaff, A. J. Miller, Improving the yield and nutritional quality of forage crops. *Front. Plant Sci.* **9**, 535 (2018).
52. O. Hoegh-Guldberg, D. Jacob, M. Taylor, T. Guillén Bolaños, M. Bindi, S. Brown, I. A. Camilloni, A. Diedhiou, R. Djalante, K. Ebi, F. Engelbrecht, J. Guiot, Y. Hijioka, S. Mehrotra, C. W. Hope, A. J. Payne, H.-O. Pörtner, S. I. Seneviratne, A. Thomas, R. Warren, G. Zhou, The human imperative of stabilizing global climate change at 1.5°C. *Science* **365**, eaaw6974 (2019).
53. Y.-M. Wei, R. Han, C. Wang, B. Yu, Q.-M. Liang, X.-C. Yuan, J. Chang, Q. Zhao, H. Liao, B. Tang, J. Yan, L. Cheng, Z. Yang, Self-preservation strategy for approaching global warming targets in the post-Paris Agreement era. *Nat. Commun.* **11**, 1624 (2020).
54. S. Kou-Giesbrecht, V. K. Arora, C. Seiler, A. Arneth, S. Falk, A. K. Jain, F. Joos, D. Kennedy, J. Knauer, S. Sitch, M. O'Sullivan, N. Pan, Q. Sun, H. Tian, N. Vuichard, S. Zaehle, Evaluating nitrogen cycling in terrestrial biosphere models: A disconnect between the carbon and nitrogen cycles. *Earth Syst. Dyn.* **14**, 767–795 (2023).
55. B. D. Stocker, N. Dong, E. A. Perkowski, P. D. Schneider, H. Xu, H. J. De Boer, K. T. Rebel, N. G. Smith, K. Van Sundert, H. Wang, S. E. Jones, I. C. Prentice, S. P. Harrison, Empirical evidence and theoretical understanding of ecosystem carbon and nitrogen cycle interactions. *New Phytol.* **245**, 49–68 (2025).
56. R. Savin, V. O. Sadras, G. A. Slafer, Benchmarking nitrogen utilisation efficiency in wheat for Mediterranean and non-Mediterranean European regions. *Field Crop Res* **241**, 107573 (2019).
57. J. Chang, P. Ciais, T. Gasser, P. Smith, M. Herrero, P. Havlík, M. Obersteiner, B. Guenet, D. S. Goll, W. Li, V. Naipal, S. Peng, C. Qiu, H. Tian, N. Viovy, C. Yue, D. Zhu, Climate warming from managed grasslands cancels the cooling effect of carbon sinks in sparsely grazed and natural grasslands. *Nat. Commun.* **12**, 118 (2021).
58. P. Xu, G. Li, Y. Zheng, J. C. H. Fung, A. Chen, Z. Zeng, H. Shen, M. Hu, J. Mao, Y. Zheng, X. Cui, Z. Guo, Y. Chen, L. Feng, S. He, X. Zhang, A. K. H. Lau, S. Tao, B. Z. Houlton, Fertilizer management for global ammonia emission reduction. *Nature* **626**, 792–798 (2024).
59. S. Pfahl, P. A. O'Gorman, E. M. Fischer, Understanding the regional pattern of projected future changes in extreme precipitation. *Nat. Clim. Change* **7**, 423–427 (2017).
60. C. Raymond, R. M. Horton, J. Zscheischler, O. Martius, A. AghaKouchak, J. Balch, S. G. Bowen, S. J. Camargo, J. Hess, K. Kornhuber, M. Oppenheimer, A. C. Ruane, T. Wahl, K. White, Understanding and managing connected extreme events. *Nat. Clim. Change* **10**, 611–621

(2020).
61. T. Davies-Barnard, P. Friedlingstein, The global distribution of biological nitrogen fixation in terrestrial natural ecosystems. *Global Biogeochem. Cycles* **34**, e2019GB006387 (2020).
62. C. C. Cleveland, A. R. Townsend, D. S. Schimel, H. Fisher, R. W. Howarth, L. O. Hedin, S. S. Perakis, E. F. Latty, J. C. Von Fischer, A. Elseroad, M. F. Wasson, Global patterns of terrestrial biological nitrogen (N_2) fixation in natural ecosystems. *Global Biogeochem. Cycles* **13**, 623–645 (1999).
63. P. M. Vitousek, D. N. L. Menge, S. C. Reed, C. C. Cleveland, Biological nitrogen fixation: Rates, patterns and ecological controls in terrestrial ecosystems. *Philos. Trans. R. Soc. B* **368**, 20130119 (2013).
64. L. Lassaletta, G. Billen, B. Grizzetti, J. Anglade, J. Garnier, 50 year trends in nitrogen use efficiency of world cropping systems: the relationship between yield and nitrogen input to cropland. *Environ. Res. Lett.* **9**, 105011 (2014).
65. P. Heffer, M. Prud'homme, "Short-term fertilizer outlook 2014–2015," in *IFA Strategic Forum Marrakech (Morocco)* (International Fertilizer Industry Association, 2014).
66. L. Liu, W. Xu, Z. Wen, P. Liu, H. Xu, S. Liu, X. Lu, B. Zhong, Y. Guo, X. Lu, Y. Zhao, X. Zhang, S. Wang, P. M. Vitousek, X. Liu, Modeling global oceanic nitrogen deposition from food systems and its mitigation potential by reducing overuse of fertilizers. *Proc. Natl. Acad. Sci. U.S.A.* **120**, e2221459120 (2023).
67. L. Bouwman, K. K. Goldewijk, K. W. Van Der Hoek, A. H. W. Beusen, D. P. Van Vuuren, J. Willems, M. C. Rufino, E. Stehfest, Exploring global changes in nitrogen and phosphorus cycles in agriculture induced by livestock production over the 1900–2050 period. *Proc. Natl. Acad. Sci. U.S.A.* **110**, 20882–20887 (2013).
68. J. A. Van Aardenne, F. J. Dentener, J. G. J. Olivier, C. G. M. K. Goldewijk, J. Lelieveld, A 1°×1° resolution data set of historical anthropogenic trace gas emissions for the period 1890–1990. *Global Biogeochem. Cycles* **15**, 909–928 (2001).
69. A. F. Bouwman, L. J. M. Boumans, N. H. Batjes, Estimation of global NH_3 volatilization loss from synthetic fertilizers and animal manure applied to arable lands and grasslands. *Global Biogeochem. Cycles* **16**, 8-1–8-14 (2002).
70. J. N. Galloway, F. J. Dentener, D. G. Capone, E. W. Boyer, R. W. Howarth, S. P. Seitzinger, G. P. Asner, C. C. Cleveland, P. A. Green, E. A. Holland, D. M. Karl, A. F. Michaels, J. H. Porter,

A. R. Townsend, C. J. Vöosmarty, Nitrogen cycles: Past, present, and future. *Biogeochemistry* **70**, 153–226 (2004).

71. B. Pan, S. K. Lam, A. Mosier, Y. Luo, D. Chen, Ammonia volatilization from synthetic fertilizers and its mitigation strategies: A global synthesis. *Agr Ecosyst Environ* **232**, 283–289 (2016).
72. S. R. S. Dangal, H. Tian, R. Xu, J. Chang, J. G. Canadell, P. Ciais, S. Pan, J. Yang, B. Zhang, Global nitrous oxide emissions from pasturelands and rangelands: Magnitude, spatiotemporal patterns, and attribution. *Global Biogeochem. Cycles* **33**, 200–222 (2019).
73. Z. Wei, E. Hoffland, M. Zhuang, P. Hellegers, Z. Cui, Organic inputs to reduce nitrogen export via leaching and runoff: A global meta-analysis. *Environ. Pollut.* **291**, 118176 (2021).
74. E. E. C. Buendia, K. Tanabe, A. Kranjc, B. Jamsranjav, M. Fukuda, S. Ngarize, A. Osako, Y. Pyrozhenko, P. Shermanau, S. Federici, *2019 Refinement to the 2006 IPCC Guidelines for National Greenhouse Gas Inventories. Volume 4: Agriculture, Forestry and Other Land Use* (IPCC, 2019).
75. I. Harris, T. J. Osborn, P. Jones, D. Lister, Version 4 of the CRU TS monthly high-resolution gridded multivariate climate dataset. *Sci. Data* **7**, 109 (2020).
76. R. J. Zomer, J. Xu, A. Trabucco, Version 3 of the global aridity index and potential evapotranspiration database. *Sci. Data* **9**, 409 (2022).
77. A. Ortiz-Bobea, T. R. Ault, C. M. Carrillo, R. G. Chambers, D. B. Lobell, Anthropogenic climate change has slowed global agricultural productivity growth. *Nat. Clim. Change* **11**, 306–312 (2021).
78. F. C. Moore, D. B. Lobell, The fingerprint of climate trends on European crop yields. *Proc. Natl. Acad. Sci. U.S.A.* **112**, 2670–2675 (2015).
79. D. B. Lobell, J. A. Burney, Cleaner air has contributed one-fifth of US maize and soybean yield gains since 1999. *Environ. Res. Lett.* **16**, 074049 (2021).
80. J. G. MacKinnon, M. Webb, "When and how to deal with clustered errors in regression models" (Queen's Economics Department Working Paper 1421, Queen's University, Department of Economics, Kingston, 2019).
81. C. E. Moody, T. B. Marvell, Clustering and standard error bias in fixed effects panel data regressions. *J. Quant. Criminol.* **36**, 347–369 (2020).
82. M. Kotz, L. Wenz, A. Stechemesser, M. Kalkuhl, A. Levermann, Day-to-day temperature

variability reduces economic growth. *Nat. Clim. Change* **11**, 319–325 (2021).

83. X. Zhang, B. Gu, H. Van Grinsven, S. K. Lam, X. Liang, M. Bai, D. Chen, Societal benefits of halving agricultural ammonia emissions in China far exceed the abatement costs. *Nat. Commun.* **11**, 4357 (2020).
84. Z. Zhu, X. Zhang, H. Dong, S. Wang, S. Reis, Y. Li, B. Gu, Integrated livestock sector nitrogen pollution abatement measures could generate net benefits for human and ecosystem health in China. *Nat. Food* **3**, 161–168 (2022).
85. H. J. M. Van Grinsven, M. Holland, B. H. Jacobsen, Z. Klimont, M. A. Sutton, W. Jaap Willems, Costs and benefits of nitrogen for Europe and implications for mitigation. *Environ. Sci. Technol.* **47**, 3571–3579 (2013).
86. D. J. Sobota, J. E. Compton, M. L. McCrackin, S. Singh, Cost of reactive nitrogen release from human activities to the environment in the United States. *Environ. Res. Lett.* **10**, 025006 (2015).
87. Adekunle, M. F., Public willingness to pay for ecosystem service functions of a peri-urban forest near Abeokuta, Ogun State, Nigeria. *J. Dev. Agric. Econ.* **4**, 45–50 (2012).
88. C. Brink, H. Van Grinsven, B. H. Jacobsen, A. Rabl, I.-M. Gren, M. Holland, Z. Klimont, K. Hicks, R. Brouwer, R. Dickens, J. Willems, M. Termansen, G. Velthof, R. Alkemade, M. Van Oorschot, J. Webb, "Costs and benefits of nitrogen in the environment," in *The European Nitrogen Assessment* (Cambridge Univ. Press, 2011).
89. B. Gu, L. Zhang, R. Van Dingenen, M. Vieno, H. J. Van Grinsven, X. Zhang, S. Zhang, Y. Chen, S. Wang, C. Ren, S. Rao, M. Holland, W. Winiwarter, D. Chen, J. Xu, M. A. Sutton, Abating ammonia is more cost-effective than nitrogen oxides for mitigating $PM_{2.5}$ air pollution. *Science* **374**, 758–762 (2021).
90. Z. Ouyang, C. Song, H. Zheng, S. Polasky, Y. Xiao, I. J. Bateman, J. Liu, M. Ruckelshaus, F. Shi, Y. Xiao, W. Xu, Z. Zou, G. C. Daily, Using gross ecosystem product (GEP) to value nature in decision making. *Proc. Natl. Acad. Sci. U.S.A.* **117**, 14593–14601 (2020).
91. A. R. Ravishankara, J. S. Daniel, R. W. Portmann, Nitrous oxide (N_2O): The dominant ozone-depleting substance emitted in the 21st century. *Science* **326**, 123–125 (2009).
92. B. Zhang, The effect of aerosols to climate change and society. *J. Geosci. Environ. Prot.* **08**, 55–78 (2020).
93. X. W. Hu, E. N. H. Lian, A study on accounting for the gross value of grassland ecosystems in Qinghai Province. *Qinghai Tibet Plateau Forum* **1**, 54–59 (2022).

94. H. Chen, R. Costanza, I. Kubiszewski, Legitimacy and limitations of valuing the oxygen production of ecosystems. *Ecosyst. Serv.* **58**, 101485 (2022).
95. L. I. Eide, M. Batum, T. Dixon, Z. Elamin, A. Graue, S. Hagen, S. Hovorka, B. Nazarian, P. H. Nøkleby, G. I. Olsen, P. Ringrose, R. A. M. Vieira, Enabling large-scale carbon capture, utilisation, and storage (CCUS) using offshore carbon dioxide (CO_2) infrastructure developments—A review. *Energies* **12**, 1945 (2019).
96. R. Costanza, B. Hannon, K. Limburg, S. Naeem, R. V. O'Neill, R. G. Raskin, P. Sutton, The value of the world's ecosystem services and natural capital. *Nature* **387**, 253–260 (1997).
97. B. Gu, J. Chang, Y. Ge, H. Ge, C. Yuan, C. Peng, H. Jiang, Anthropogenic modification of the nitrogen cycling within the greater Hangzhou area system, China. *Ecol. Appl.* **19**, 974–988 (2009).
98. K. Riahi, D. P. Van Vuuren, E. Kriegler, J. Edmonds, B. C. O'Neill, S. Fujimori, N. Bauer, K. Calvin, R. Dellink, O. Fricko, W. Lutz, A. Popp, J. C. Cuaresma, S. Kc, M. Leimbach, L. Jiang, T. Kram, S. Rao, J. Emmerling, K. Ebi, T. Hasegawa, P. Havlik, F. Humpenöder, L. A. Da Silva, S. Smith, E. Stehfest, V. Bosetti, J. Eom, D. Gernaat, T. Masui, J. Rogelj, J. Strefler, L. Drouet, V. Krey, G. Luderer, M. Harmsen, K. Takahashi, L. Baumstark, J. C. Doelman, M. Kainuma, Z. Klimont, G. Marangoni, H. Lotze-Campen, M. Obersteiner, A. Tabeau, M. Tavoni, The shared socioeconomic pathways and their energy, land use, and greenhouse gas emissions implications: An overview. *Glob. Environ. Change* **42**, 153–168 (2017).
99. B. Bajželj, K. S. Richards, J. M. Allwood, P. Smith, J. S. Dennis, E. Curmi, C. A. Gilligan, Importance of food-demand management for climate mitigation. *Nat. Clim. Change* **4**, 924–929 (2014).
